# Construction and immunohistochemical validation of a necroptosis-related prognostic signature in bladder cancer and its association with tumor immune infiltration

**DOI:** 10.3389/fgene.2025.1527907

**Published:** 2025-08-14

**Authors:** Tao Wang, Fei Ding, Kai Sun

**Affiliations:** ^1^ Department of Oncology, Lanzhou University Second Hospital, Lanzhou, China; ^2^ Department of Obstetrics and Gynecology, Gansu Provincial Maternal and Child Healthcare Hospital, Lanzhou, China; ^3^ Department of Oncology, Ganzhou Cancer Hospital, Ganzhou, Jiangxi, China

**Keywords:** bladder urothelial carcinoma, necroptosis-related, prognostic signature, tumor microenvironment infiltration, immunohistochemical validation

## Abstract

**Background:**

Bladder urothelial carcinoma (BLCA) represents a highly malignant neoplasm with significant clinical challenges. Necroptosis, a programmed form of cell death, exhibits dual regulatory functions in both tumor immunomodulation and oncogenesis. The precise mechanistic involvement of necroptosis-related genes (NRGs) in BLCA pathogenesis remains poorly characterized, prompting our systematic investigation of their potential biological and clinical significance.

**Methods and results:**

We performed comprehensive bioinformatics analyses utilizing integrated datasets from The Cancer Genome Atlas (TCGA) and the Gene Expression Omnibus (GEO) database. Through the Kyoto Encyclopedia of Genes and Genomes (KEGG) pathway annotation, we curated 159 NRGs and subsequently identified 25 differentially expressed genes functionally implicated in necrotic cell death and extrinsic apoptotic pathways, specifically including influenza A signaling, NOD-like receptor cascades, and related biological processes. Univariate Cox proportional hazards modeling coupled with LASSO regression analysis revealed five prognostically significant NRGs (*CAMK2A, CHMP4C, IL33, IRF9,* and *TRAF5*). Based on these genes, we developed a robust prognostic model that can stratify patients into high- and low-risk categories, each exhibiting distinct survival outcomes. This model demonstrated moderate accuracy in prognosis prediction. Immunohistochemical validation in BLCA specimens confirmed dysregulated expression patterns of these five NRGs. Additional analyses uncovered significant correlations between NRG expression profiles and various immunological parameters, including immune cell infiltration patterns and immune checkpoint molecule expression.

**Conclusion:**

Our study delineates a novel five-gene NRG signature with robust prognostic value in BLCA. These gene determinants appear to critically influence both tumor progression and immune microenvironment, thereby representing promising candidates for therapeutic targeting and future mechanistic exploration in bladder cancer biology.

## Introduction

Bladder urothelial carcinoma (BLCA) is a prevalent and aggressive form of cancer that presents significant challenges in terms of treatment and prognosis (Dyrskjøt et al., 2023). In recent years, significant progress has been made in the treatment of BLCA, particularly in managing muscle-invasive bladder cancer (MIBC) and metastatic bladder cancer (Lopez-Beltran et al., 2024). Traditionally, radical cystectomy has been the standard treatment for MIBC. However, it should be noted that with the diversification of treatment options, bladder-preserving approaches have gained increasing attention (Tholomier et al., 2020). The three-step therapy (TMT), which combines transurethral resection of bladder tumor (TURBT) with radiotherapy and chemotherapy, has been shown to offer comparable survival rates to radical cystectomy in appropriately selected patients, while preserving bladder function (Lopez-Beltran et al., 2024). Neoadjuvant chemotherapy is typically recommended for patients with muscle-invasive bladder cancer to shrink tumors before surgery, thus improving the thoroughness of surgical resection (Dyrskjøt et al., 2023; Lopez-Beltran et al., 2024). Postoperative adjuvant chemotherapy is also widely used for high-risk patients, especially those with a pathological stage of T3 or T4 and/or lymph node-positive (Leow et al., 2019). Cisplatin-based chemotherapy has been the leading combination treatment for BLCA for several years, significantly increasing 5-year overall survival rates in patients sensitive to chemotherapy (Li et al., 2023). Targeted therapy has also emerged as a promising approach in the treatment of BLCA. Drugs targeting fibroblast growth factor receptor (FGFR), such as erdafitinib, have been approved for treating BLCA patients with specific gene mutations (Ascione et al., 2023). These targeted therapies offer new options for patients who are not responsive to traditional chemotherapy.

The tumor immune microenvironment (TIME) of BLCA is crucial for the disease’s development, progression, and treatment. Research indicates that the characteristics of the TIME can significantly influence the response to immunotherapy and overall prognosis in BLCA patients (Tan et al., 2023). The type and quantity of tumor-infiltrating immune cells (TIICs) in BLCA are closely linked to patient survival rates (Kamitani et al., 2024). The TIME of BLCA typically exhibits a high degree of heterogeneity, which is evident not only among different patients but also across different tumor regions within the same patient (Luo et al., 2024). For example, studies have shown that the patterns of immune cell infiltration in BLCA can be categorized into three subtypes: immune rejection, immune inflammation, and immune desert (Huang et al., 2021). These subtypes are closely linked to the patient’s prognosis and response to immune checkpoint inhibitors (ICIs). Moreover, DNA repair defects (DDR) in BLCA are also considered an important factor affecting the efficacy of immunotherapy. Studies have shown that patients with *ATM* gene mutations in BLCA show better efficacy when treated with immune checkpoint inhibitors, which may be related to their higher mutation load and immunogenicity (Luo et al., 2024; Xu et al., 2024). The TIME of BLCA is also influenced by other factors in the tumor microenvironment (TME), such as hypoxia and changes in metabolic pathways. Hypoxia can alter the TIME by affecting the function and infiltration of immune cells, thereby impacting the effectiveness of immunotherapy (Chen et al., 2023). Additionally, metabolic-related gene characteristics are closely linked to the immune microenvironment and prognosis of BLCA patients (Guo et al., 2023). The introduction of immunotherapy has brought new hope to the treatment of BLCA. Immunotherapy agents, such as PD-1 and PD-L1 inhibitors, have shown significant efficacy in treating metastatic bladder cancer and have been approved for first-line and second-line treatments (Khandakar et al., 2025). These drugs can provide durable responses in some patients, although the overall response rate remains limited (Konala et al., 2022; Petrelli et al., 2022). In conclusion, the tumor immune microenvironment of BLCA is a complex and dynamic system, and its characteristics and changes have an important impact on patients' treatment response and prognosis. An in-depth study of the composition and function of TIME will help develop more effective personalized immunotherapy strategies.

Cell death is crucial for the homeostasis, growth, and development of multicellular organisms. Most human diseases stem from irregularities in these processes. Among various cell death types, necroptosis is a distinct, regulated form of programmed cell death (Zhu and Wu, 2024). Necroptosis is a vital form of programmed cell death marked by unique features such as cell membrane rupture, organelle swelling, disintegration of cytoplasm and nucleus, leakage of cellular contents, release of damage-associated molecular patterns (DAMPs), and inflammatory responses (Bertheloot et al., 2021; Meier et al., 2024; Zhu and Wu, 2024). In contrast to apoptosis, which is a caspase-dependent, non-inflammatory, and tightly regulated process, necroptosis operates via a caspase-independent pathway and culminates in cell death with pro-inflammatory outcomes (Gielecińska et al., 2023). This inflammatory aspect aligns necroptosis more closely with necrosis, traditionally perceived as an uncontrolled response to acute cellular injury (Otręba et al., 2023). Nevertheless, necroptosis is a regulated process, distinguishing it from the accidental nature of necrosis. The molecular framework of necroptosis involves critical proteins such as receptor-interacting protein kinase 1 (RIPK1), RIPK3, and mixed lineage kinase domain-like (MLKL) protein (Yuan et al., 2019; Martens et al., 2021). Upon activation, RIPK1 and RIPK3 assemble into a complex termed the necrosome, which subsequently activates MLKL. Once phosphorylated, MLKL translocates to the plasma membrane, resulting in membrane permeabilization and eventual cell rupture, thereby releasing DAMPs that incite inflammation (Chen et al., 2019). In contrast, apoptosis is distinguished by cell shrinkage, chromatin condensation, and DNA fragmentation, with cellular contents being encapsulated into apoptotic bodies that are subsequently phagocytosed by adjacent cells, thereby avoiding the induction of an inflammatory response. This process is predominantly facilitated by caspases, a family of cysteine proteases that are not implicated in necroptosis (Fritsch et al., 2019; Gielecińska et al., 2023). Necroptosis, on the other hand, can be initiated by various stimuli, including tumor necrosis factor (TNF), Fas ligand, and certain viral infections, particularly in contexts where caspase activity is inhibited or absent (Ye et al., 2023; Peng, 2024). This pathway functions as a compensatory mechanism to ensure cell death when apoptosis is obstructed, underscoring its role as a fail-safe mechanism in maintaining cellular homeostasis and modulating immune responses (Meier et al., 2024). Moreover, necroptosis has been associated with a range of pathological conditions, including inflammatory diseases, neurodegeneration, and cancer (Yin et al., 2024). Its capacity to induce inflammation renders it a double-edged sword, with the potential to either contribute to disease progression or act as a defense mechanism against pathogens (Ye et al., 2023; Zhang R. et al., 2023; Zhao et al., 2023).

In the realm of oncological therapeutics, necroptosis emerges as a promising target for circumventing resistance to apoptosis, a prevalent obstacle in cancer treatment (Meier et al., 2024). The capacity of necroptosis to induce immunogenic cell death positions it as a viable strategy for augmenting antitumor immunity (Yin et al., 2024). This is particularly pertinent in malignancies where apoptotic pathways are compromised, thereby allowing necroptosis to function as an alternative mechanism to induce cell death and stimulate an immune response (Zhang J. et al., 2022; 2024). Furthermore, necroptosis has been implicated in the modulation of the TME, which is instrumental in cancer progression and therapeutic response (Zhang et al., 2024). The release of DAMPs during necroptosis can activate the immune system and modify the TME, potentially enhancing the efficacy of immunotherapies (Yu et al., 2024). Consequently, there is growing interest in exploring necroptosis-related genes as prognostic biomarkers and therapeutic targets in various cancers (Ding et al., 2022; Zhang T. et al., 2022). In addition to its involvement in cancer progression, necroptosis is currently under investigation for its potential to enhance the efficacy of existing cancer therapies (Zhu et al., 2022). By inducing necroptosis, researchers seek to augment the immunogenicity of tumor cells, thereby enhancing the response to immunotherapies and diminishing the likelihood of treatment resistance (Guo et al., 2025). This strategy holds particular promise for cancers characterized by a high tumor mutation burden (TMB) and those employing immune evasion tactics (Meier et al., 2024; Yin et al., 2024; Guo et al., 2025). The exploration of necroptosis in cancer is advancing rapidly, with ongoing research dedicated to elucidating its molecular mechanisms and therapeutic potential. Targeting necroptosis pathways offers the potential to develop novel cancer treatments capable of overcoming the limitations of current therapies and improving patient outcomes (Zang et al., 2022; Yin et al., 2024).

This study aims to fill this gap by examining the role of necroptosis in the progression of BLCA and its interactions with the tumor immune microenvironment, thereby advancing the understanding of diagnostic and prognostic markers for the disease. Utilizing data from the TCGA database, we explored the relevance of NRGs in predicting the prognosis of BLCA. A necroptosis risk-scoring prognostic signature was developed based on the identified prognostic NRGs. The applicability and prognostic value of this predictive model were subsequently validated. Additionally, we examined the expression levels of the candidate NRGs in 22 BLCA tissues and their matched adjacent normal tissues using immunohistochemistry (IHC). We also analyzed the relationship between NRGs and the immune microenvironment in BLCA. This study may provide valuable insights into potential diagnostic and prognostic biomarkers for BLCA.

## Materials and methods

### Datasets and data processing

The UCSC Xena dataset was used to acquire TCGA and the Genotype-Tissue Expression (GTEx) expression and clinical information (https://toil-xena-hub.s3.us-east-1.amazonaws.com/download/TcgaTargetGtex_rsem_gene_tpm.gz; Full metadata) (Goldman et al., 2020). Dataset ID: TcgaTargetGtex_rsem_gene_tpm. Raw counts of RNA-sequencing data (level 3) and clinical data matching were downloaded from the TCGA and GTEx databases. There are 408 BLCA samples and 40 normal bladder samples (19 from the TCGA and 21 from GTEx) (Table 1). To strengthen the reliability of our results, we augmented our dataset by incorporating multiple BLCA cohorts obtained from the Gene Expression Omnibus (GEO, https://www.ncbi.nlm.nih.gov/geo/). These cohorts include GSE13507 (70 BLCA samples), GSE19423 (48 BLCA samples), GSE37815 (24 BLCA samples), GSE48075 (142 BLCA samples, including 73 advanced BLCA samples), GSE69795 (61 BLCA samples), and GSE154261 (99 BLCA samples) (Barrett et al., 2013). We also included the IMvigor210 cohort (348 BLCA samples) sourced from the Tumor Immunotherapy Gene Expression Resource (TIGER) database. A total of 159 NRGs were retrieved from the KEGG database (Kanehisa and Goto, 2000). R software version 4.0.3 was used for all analyses. Following the extraction of data in the TPM format, normalization was performed utilizing a logarithmic transformation, specifically log2 (TPM +1). Missing data within the databases were imputed using the missForest package in R (Stekhoven and Bühlmann, 2012).

**TABLE 1:** Clinical characteristics of patients with BLCA.

Characteristic	levels	Overall
n		408
T stage, n (%)	T1	3 (0.8%)
	T2	119 (31.8%)
	T3	194 (51.9%)
	T4	58 (15.5%)
N stage, n (%)	N0	237 (64.8%)
	N1	46 (12.6%)
	N2	75 (20.5%)
	N3	8 (2.2%)
M stage, n (%)	M0	196 (94.7%)
	M1	11 (5.3%)
Age, median (IQR)		69 (60, 76)

### Tissue samples and immunohistochemistry

A total of 22 BLCA tumor samples and 22 matched adjacent normal tissues from the same patients (total n = 44 specimens) were collected in Liuzhou People’s Hospital. The Ethics Committee of Liuzhou People’s Hospital approved the project (Reference No. KY2022-035-01), and we conducted it according to the Declaration of Helsinki. Relevant clinical data can be found in Supplementary Table S1. Three pathologists examined all tissues pathologically. Slices were dewaxed and hydrated, followed by antigen retrieval. Following the blocking step by endogenous peroxide blockers and normal goat serum, slices were incubated with primary CAMK2A antibody (1:500; Sino Biological, China), anti-CHMP4C (1:100; Cusabio, China), anti-IL33 (1:200; Sino Biological, China), anti-IRF9 (1:200; Proteintech, China), and anti-TRAF5 (1:50; Sangon Biotech, China), as well as horseradish peroxidase-conjugated secondary antibodies (Maxim, China). Then, slices underwent DAB and hematoxylin staining. The integrated optical density (IOD) of each slice was measured by Image Pro Plus 6.0 software.

### Differential expression analysis, mutation analysis, survival analysis, and correlation analysis

We investigated the differentially expressed NRGs between BLCA tumor tissue and normal tissue via R packages “DESeq2” and “limma.” The cut-off criteria were adjusted *P*-values <0.05 and |Log2-fold change | > 1 (Liu et al., 2021). The Venn diagram was also drawn. The complex heatmap, volcano plot, boxplot, expression analysis, and survival curves of the comparison between normal tissues and tumor tissues were drawn using the R packages “complex Heatmap,” “volcano,” “boxplot,” “ggplot2,” “survival,” and “survminer” (Ito and Murphy, 2013). Kaplan–Meier survival curves and the log-rank test, as well as the Cox proportional hazard regression model, were used for survival analysis. The R package “Maftools” was used to generate mutation oncoplot waterfalls and frequency plots for 25 NRGs in BLCA patients (Mayakonda et al., 2018). Pearson correlation’s analysis or Spearman’s correlation analysis was used to examine the relationship between quantitative variables.

### Functional enrichment analysis

We used the R package “ClusterProfiler (Yu et al., 2012)” to carry out Gene Ontology (GO) enrichment analyses, including the biological process (BP), cellular component (CC), and molecular function (MF) categories and the KEGG pathways of co-expression genes. Results were visualized using the R package “ggplot2.”

### Establishment of a necroptosis risk-scoring signature for prognosis

We evaluated NRG prognostic significance using Cox regression analysis. Kaplan–Meier survival curves were built, and hazard ratios (HR) with 95% confidence interval (CI) were calculated by log-rank tests. The optimal penalty parameter (λ) was determined via 10-fold cross-validation based on the minimum partial likelihood deviance (λ.min = 0.022) and the 1-SE rule (λ.1se = 0.032) to maximize parsimony. The signature genes selected at λ.1se were entered into a multivariate Cox proportional hazards model. Only variables achieving statistical significance (*P* < 0.05) were retained in the final prognostic model. As a result, we selected five significant prognostic value NRGs for further analysis. Then, we constructed the prognostic model based on these five prognostic NRGs by LASSO Cox regression analysis (Zhang et al., 2020). According to the median risk score, TCGA patients with BLCA were divided into two subgroups, low- and high-risk, and the KM analysis time was compared to the overall survival (OS) between the two subgroups. We predicted the accuracy of each gene and the risk score via time receiver operating characteristic (ROC) analysis. A nomogram to quantitatively predict 1-, 3-, and 5-year overall survival was developed using the clinical characteristics (Jeong et al., 2020). An R package called “forestplot” was used to visualize the *P*-values, HR, and 95% CI of each variable.

### Immune correlation analysis

The R packages “GSVA,” “immunedeconv,” “estimate,” “ggplot2,” “pheatmap,” and “ggstatsplot” were used to analyze the correlation between these five prognostic NRGs and immune cell infiltration. Three of the latest algorithms, including ssGSEA, ESTIMATE, and CIBERSORT, were also used. Stromal, immune, and estimate scores, 60 common immune checkpoint molecules, and 150 marker genes were identified for five immune pathways (Ohtani, 2007; Chen et al., 2018; 2022). The statistical analysis information was visualized using R version 4.0.3.

### Statistical analysis

An analysis of the data was conducted using a log-rank test, including fold change (FC), HR, and *P*-values. The strength of the relationships between variables was determined by Spearman’s correlation analysis or Pearson correlation analysis, with r values as the measure of correlation. Results were considered to be statistically significant when the *P*-value or log-rank *P*-value was less than 0.05.

## Results

### Identification of necroptosis-related genes in patients with BLCA

**FIGURE 1:**
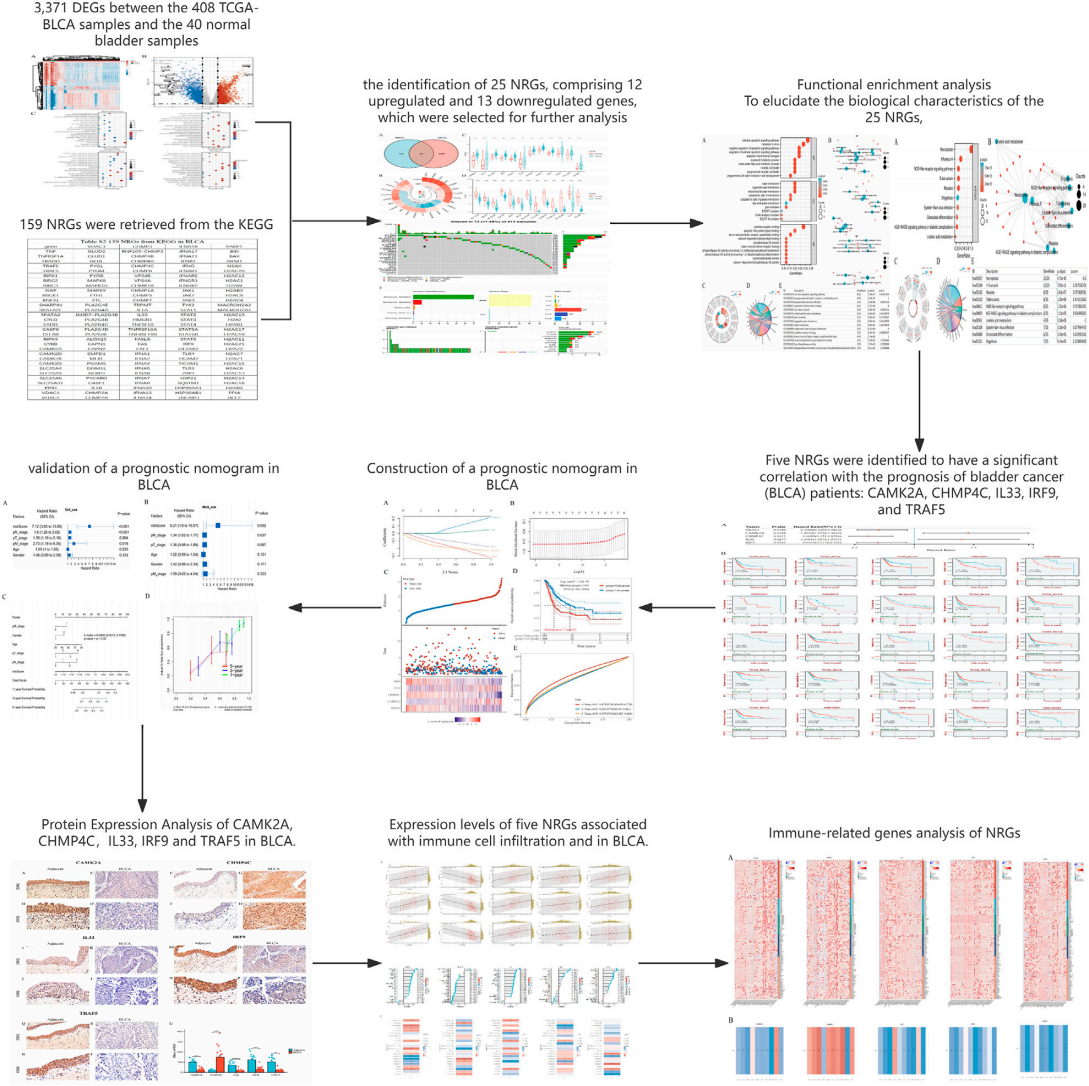
The workflow of the present study.

**FIGURE 2:**
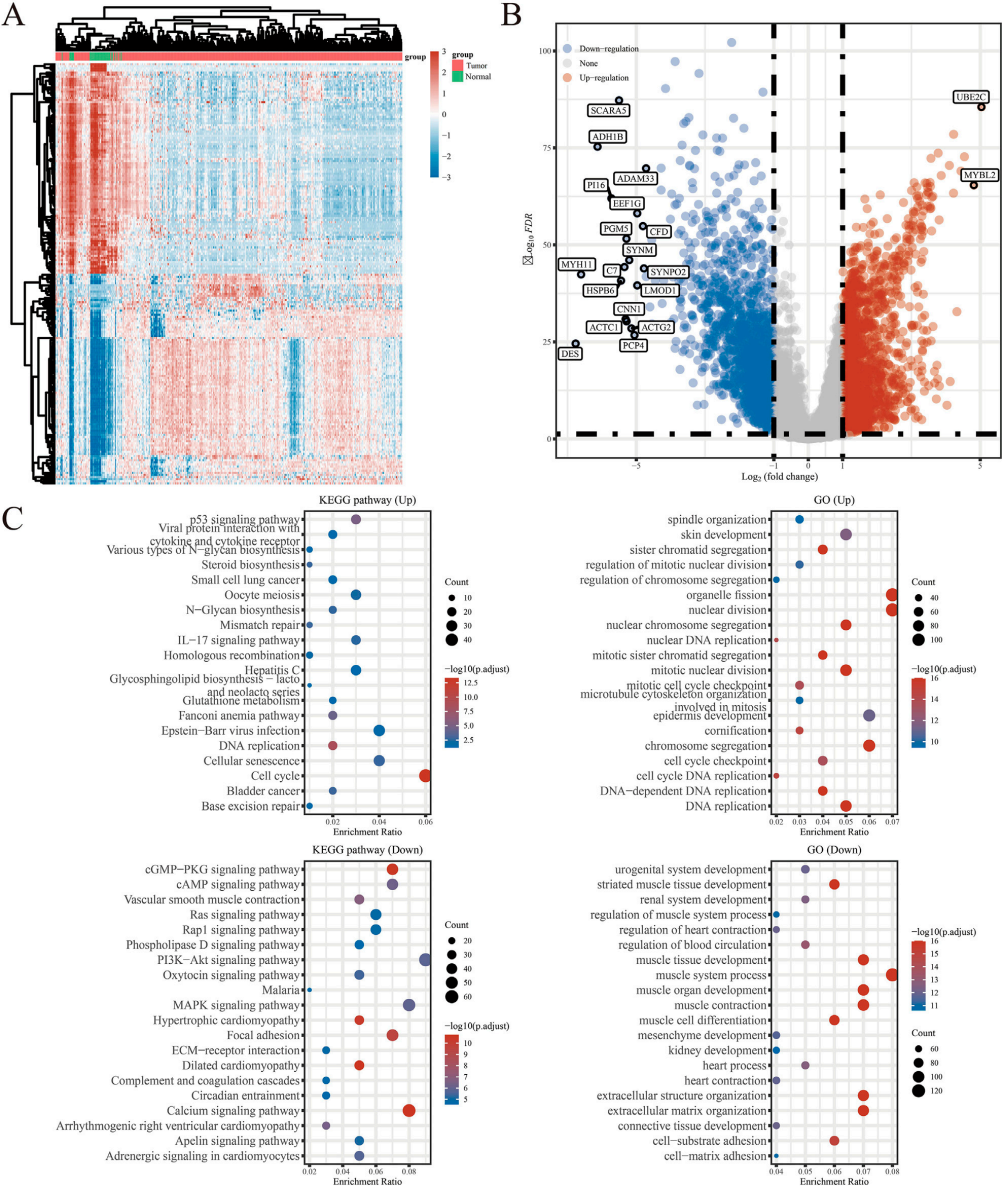
**(A)** Heatmap of the expression levels of 3371 DEGs in BLCA. **(B)** Volcano plot of the expression levels of 3371 DEGs in BLCA. **(C)** Enriched Gene Ontology terms and KEGG pathways associated with the 3371 DEGs in BLCA. *P < 0.05, **P < 0.01, and ***P < 0.001 Abbreviations: DEGs, differentially expressed genes; BLCA, bladder urothelial carcinoma; KEGG, Kyoto Encyclopedia of Genes and Genomes; GO: Gene Ontology.

**FIGURE 3:**
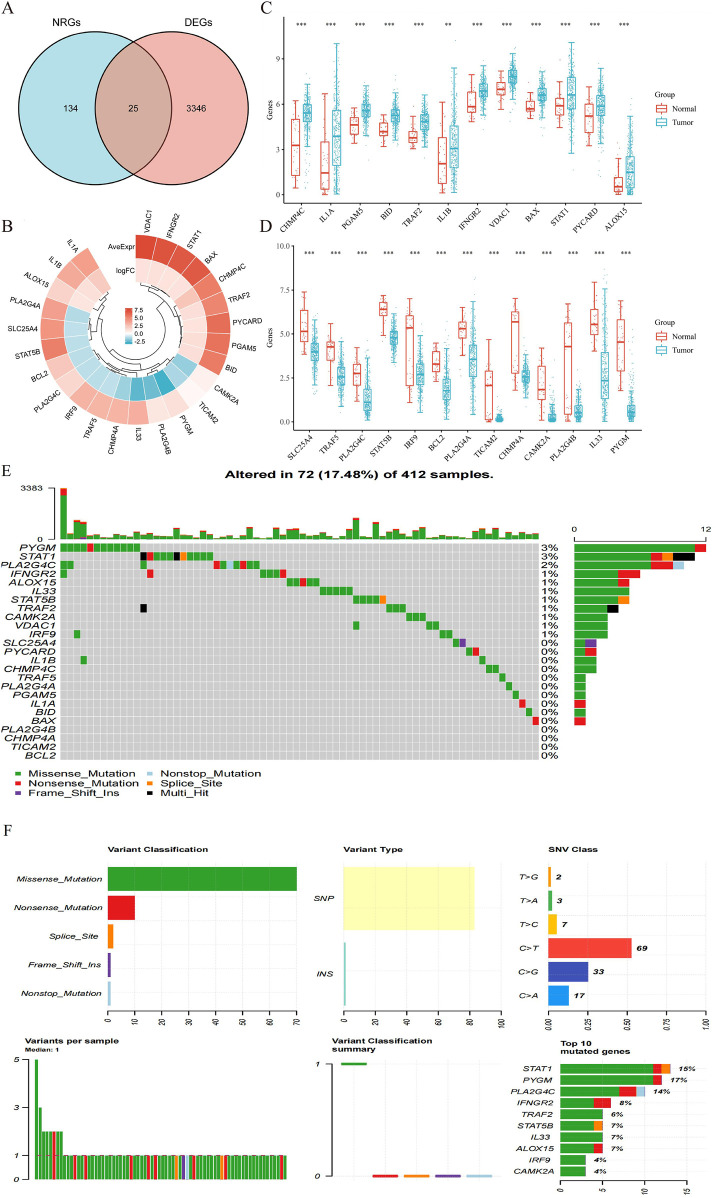
**(A)** Venn diagram of the intersection of NRGs and DEGs. **(B)** A total of 25 NRGs were found in the DEGs between BLCA and normal samples. **(C,D)** The expression of 25 NRGs in BLCA and normal bladder tissues; Normal, red; Tumor, blue. Abbreviations: BLCA, bladder urothelial carcinoma; DEGs, differentially expressed genes; NRGs, necroptosis-related genes. **(E)** Frequency of genetic alterations in 412 samples. **(F)** Variant classification and type details and summary of top 10 mutated genes with their mutation rates.

The detailed flowchart of the study is presented in Figure 1. Gene expression profiles from 408 BLCA tumor samples and 40 normal bladder samples from patients without BLCA were obtained from TCGA and GTEx projects. Additionally, survival and clinical data were acquired, excluding patients who were lost to follow-up. A total of 159 NRGs were retrieved from the Kyoto Encyclopedia of Genes and Genomes (KEGG) (Supplementary Table S2). Differentially expressed genes (DEGs) were identified using cut-off criteria of |log2-fold change| > 1 and an adjusted *P*-value <0.05. Through overlapping analyses using limma, edgeR, and DESeq2, we identified 3,371 DEGs between the 408 TCGA-BLCA samples and the 40 normal bladder samples (Figure 2). The expression levels of each gene across the 448 specimens were visualized using a heat map and a volcano plot (Figures 2A,B). Enrichment analyses were conducted to investigate the functional roles of these genes in BLCA (Figure 2C). Subsequently, the intersection of 3,371 upregulated and downregulated DEGs with 159 necroptosis-related genes resulted in the identification of 25 NRGs, comprising 12 upregulated and 13 downregulated genes, which were selected for further analysis (Figures 3A–D) (Supplementary Table S3). Consequently, we conducted an analysis and synthesis of the somatic mutations present in 25 identified NRGs within BLCA samples. The findings revealed that 72 of 412 BLCA samples (17.48%) exhibited genetic mutations, as depicted in Figures 3E,F. Missense mutations emerged as the predominant variant classification (Figure 3E). Single-nucleotide polymorphisms (SNPs) were identified as the most prevalent variant type, with the C > T transition being the most common single-nucleotide variation (SNV) class (Figure 3F). Consequently, the genes *STAT1, PYGM, PLA2G4C, IFNGR2, TRAF2, STAT5B, IL33, ALOX15, IRF9,* and *CAMK2A* were identified as the top ten NRGs with the highest mutation frequency among the 25 genes analyzed.

### Functional enrichment analysis

**FIGURE 4:**
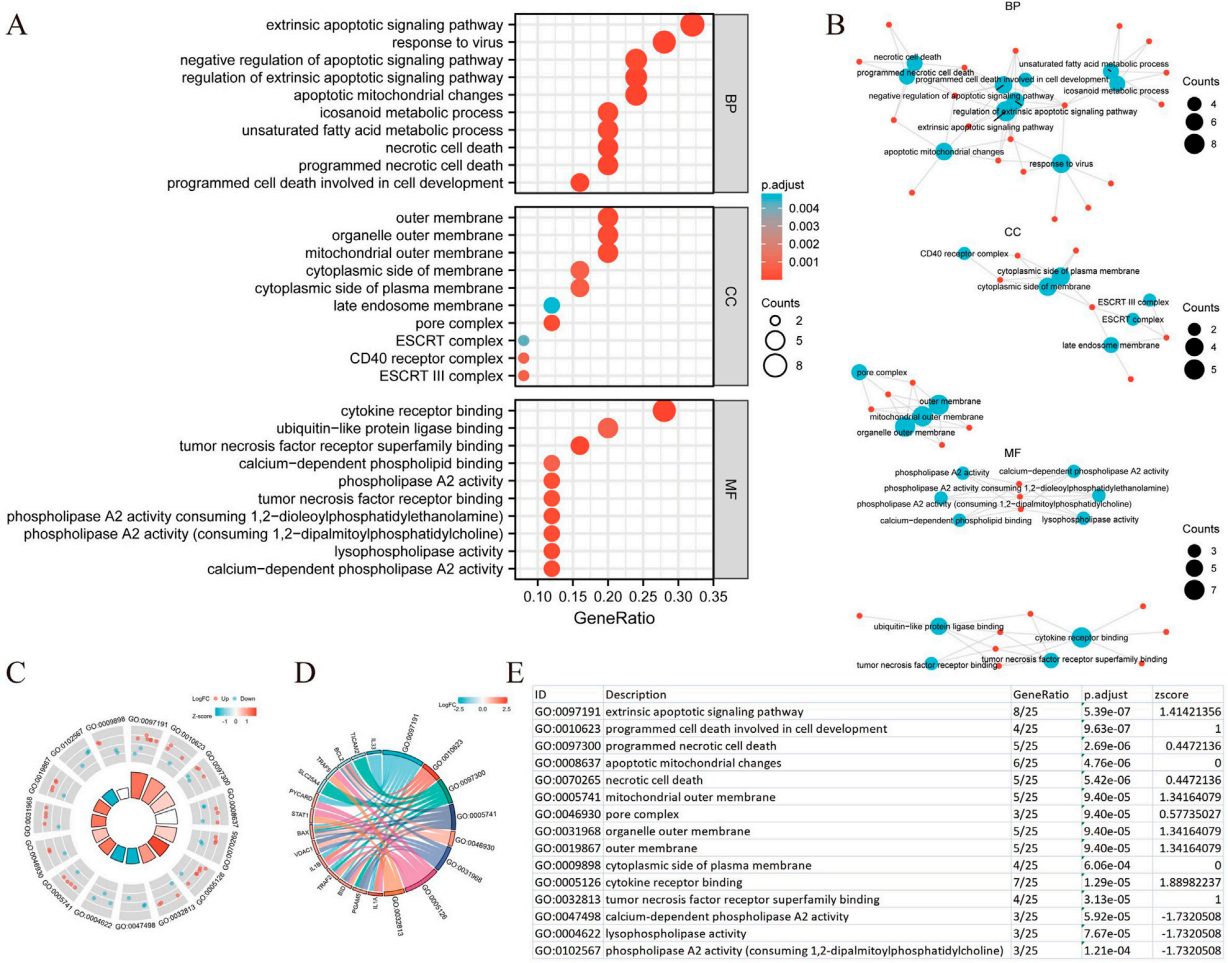
GO analysis of differentially expressed NRGs in BLCA. (A) The significant terms of GO function enrichment. (B) Network diagram: blue nodes represent items, red nodes represent molecules, and the lines represent the relationships between items and molecules. (C) The GO circle shows a scatter map of the specified gene’s logFC. (D) Enrichment string diagrams of NRGs. (E) Enrichment analysis network diagram with a description of the pathways. Abbreviations: BLCA, bladder urothelial carcinoma; GO, Gene Ontology; BP, biological process; CC, cellular component; MF, molecular function; NRGs, necroptosis-related genes.

**FIGURE 5 F5:**
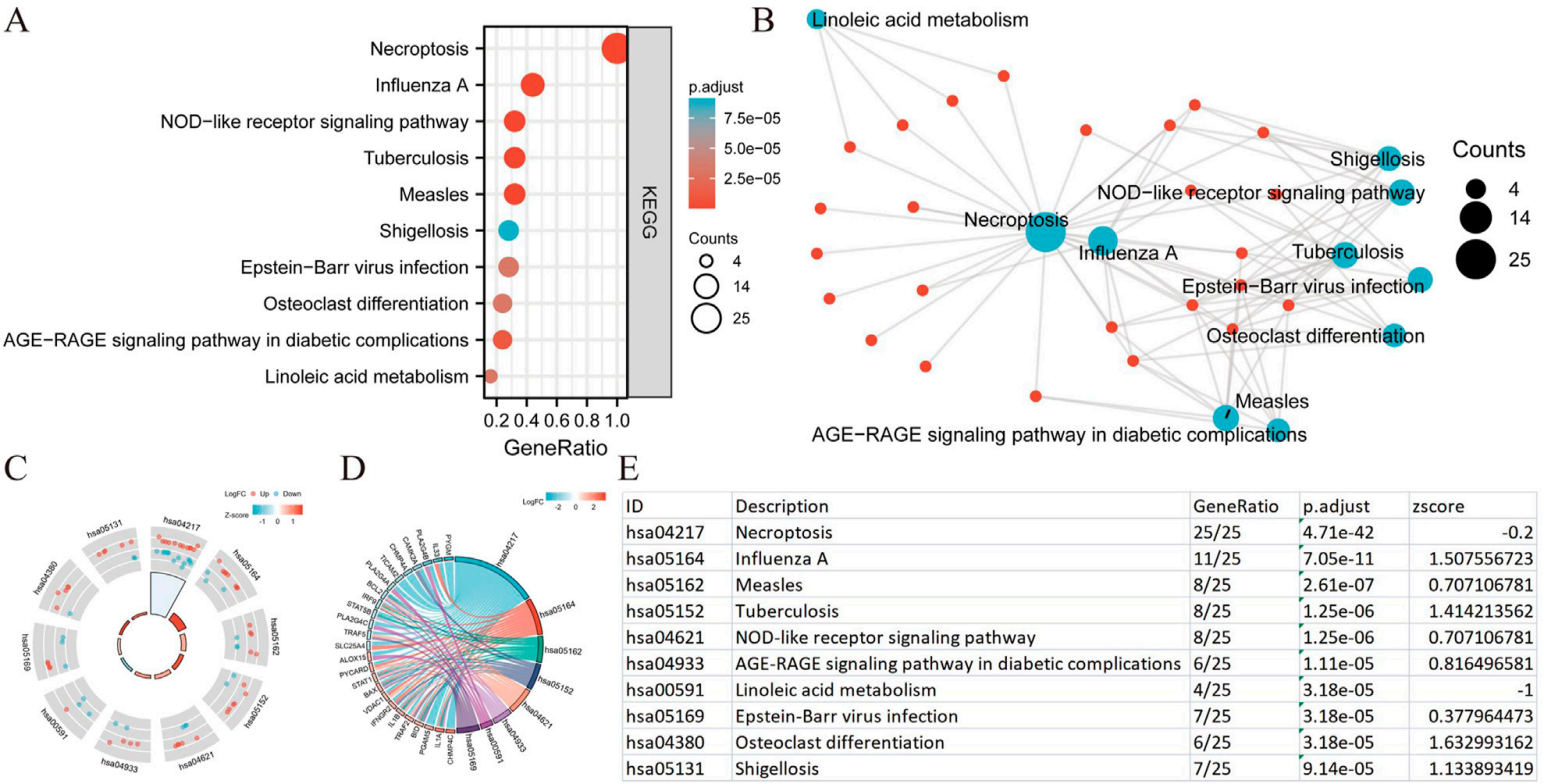
KEGG analysis of differentially expressed NRGs in BLCA. (A) The significant terms of KEGG analysis. (B) Network diagram: blue nodes represent items, red nodes represent molecules, and the lines represent the relationships between items and molecules (C) The KEGG circle shows a scatter map of the specified gene’s logFC. (D) Enrichment string diagrams of NRGs. (E) Enrichment analysis network diagram, description of pathways. Abbreviations: BLCA, bladder urothelial carcinoma; KEGG, Kyoto Encyclopedia of Genes and Genomes; NRGs, necroptosis-related genes.

We conducted a functional enrichment analysis to elucidate the biological characteristics of the 25 NRGs. The outcomes of the gene functional enrichment analysis, including Gene Ontology (GO) term enrichment and KEGG pathway analysis, are summarized in Figures 4,5 [Fig F5]. The most significantly enriched GO terms for biological processes (BP) included extrinsic apoptotic signaling, response to viruses, negative regulation of apoptosis, regulation of extrinsic apoptotic signaling, and apoptotic mitochondrial changes. The cellular components (CC) associated with these genes were identified as the outer membrane, organelle outer membrane, mitochondrial outer membrane, cytoplasmic side of the membrane, and cytoplasmic side of the plasma membrane. In terms of molecular function (MF), the enriched terms included ubiquitin-like protein ligase binding, cytokine receptor binding, tumor necrosis factor receptor superfamily binding, calcium-dependent phospholipid binding, and phospholipase A2 activity (Figures 4A,B). The KEGG pathway enrichment analysis revealed that the 25 NRGs were involved in pathways such as necroptosis, NOD-like receptor signaling, influenza A, tuberculosis, and measles (Figures 5A,B). Subsequently, we integrated these findings with Z-score analysis to predict the functional roles of the 25 NRGs within these pathways (Figures 4C,D,5C-F [Fig F5]).

### The prognostic significance of necroptosis-related genes in BLCA

**FIGURE 6:**
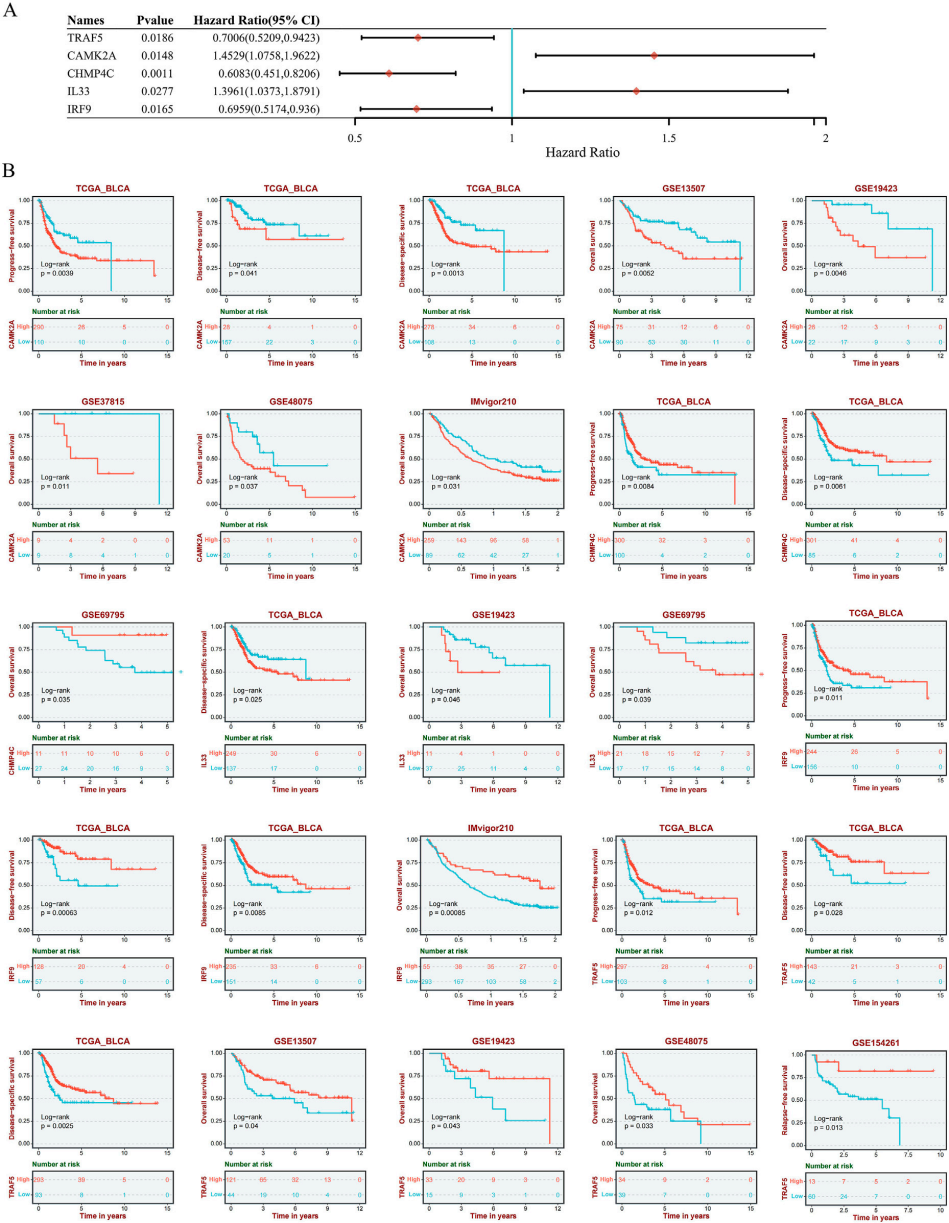
**(A)** Univariate Cox regression analysis of five NRGs in patients with BLCA in the TCGA database. **(B)** Survival analysis of five NRGs in BLCA based on the GEO and TIGER database. Abbreviations: NRGs, necroptosis-related genes; BLCA, bladder urothelial carcinoma; OS, overall survival; PFS, progression-free survival; DFS, disease-free survival; DSS, disease-specific survival; TIGER, Tumor Immunotherapy Gene Expression Resource database; TCGA, The Cancer Genome Atlas.

To evaluate the prognostic significance of 25 identified NRGs, a univariate Cox regression analysis was conducted (Supplementary Table S4). Five NRGs were identified to have a significant correlation with the prognosis of BLCA patients: *CAMK2A, CHMP4C, IL33, IRF9,* and *TRAF5* (Supplementary Table S5; Figure 6). Notably, *CHMP4C* exhibited a significant protective effect (HR = 0.6083, *P* = 0.0011), while *IRF9* and *TRAF5* also demonstrated a protective role (HR = 0.6959, *P* = 0.0165; HR = 0.7006, *P* = 0.0186). In contrast, *CAMK2A* (HR = 1.4529, *P* = 0.0148) and *IL33* (HR = 1.3961, *P* = 0.0277) were identified as risk factors (Figure 6A). Survival curve analyses across multiple databases further corroborated these findings (Figure 6B). Elevated *CAMK2A* expression correlated with poorer progression-free survival (PFS) (*P* = 0.0039), disease-free survival (DFS) (*P* = 0.041) and disease-specific survival (DSS) (*P* = 0.0039) in TCGA-BLCA and poorer overall survival (OS) in multiple validation cohorts, including GSE13507 (*P* = 0.0052), GSE19423 (*P* = 0.046), GSE37815 (*P* = 0.011), GSE48075 (*P* = 0.037), and IMvigor210 (*P* = 0.031). Conversely, a high expression of *CHMP4C* was associated with improved PFS (*P* = 0.0084) and DSS (*P* = 0.0061) in TCGA-BLCA and improved OS in GSE69795 (*P* = 0.035). IL33 overexpression predicted worse DSS in TCGA-BLCA (*P* = 0.025) and reduced OS in GSE19423 (*P* = 0.046) and GSE69795 (*P* = 0.039). *IRF9* demonstrated protective effects with improved PFS (*P* = 0.022), DFS (*P* = 0.00063), and DSS (*P* = 0.0085) in TCGA-BLCA and better OS in IMvigor210 (*P* = 0.00085). Similarly, the TRAF5 high-expression group showed improved PFS (*P* = 0.022), DFS (*P* = 0.00063), and DSS (*P* = 0.0085) in TCGA-BLCA and better prognosis in GSE13507 (*P* = 0.04), GSE19423 (*P* = 0.043), GSE48075(*P* = 0.033), and GSE154261 *(P* = 0.013). These results indicated that *CHMP4C, TRAF5*, and *IRF9* may serve as protective prognostic markers, while *CAMK2A* and *IL33* function as risk markers in BLCA.

### Construction and validation of a prognostic nomogram in BLCA

**FIGURE 7:**
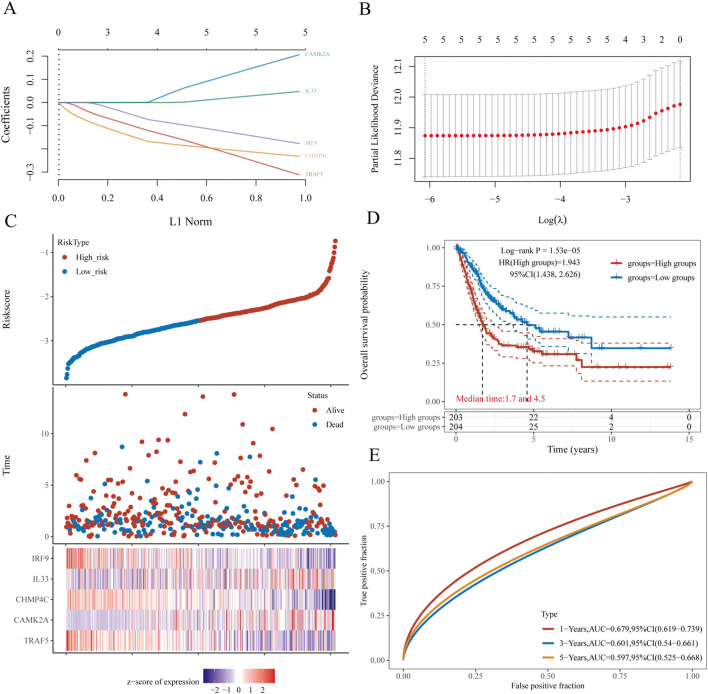
Construction and validation of a prognostic nomogram in BLCA. **(A)** LASSO coefficient profiles of five NRGs. **(B)** Plots of the ten-fold cross-validation error rates. **(C)** Distribution of risk score, survival status, and the expression of five prognostic NRGs in BLCA. **(D)** Overall survival curves for BLCA patients in the high- and low-risk groups. **(E)** The ROC curve measures the predictive value. *P < 0.05, **P < 0.01, and ***P < 0.001. Abbreviations: NRGs, necroptosis-related genes; BLCA, bladder urothelial carcinoma; LASSO, least absolute shrinkage and selection operator; ROC, receiver operating characteristic.

The selection of the five genes constituting the signature was informed by the outcomes of the LASSO regression analysis, with optimal model fitting achieved when the penalty coefficient was set to five, as illustrated in Figures 7A,B. Subsequently, a multivariate Cox regression analysis was conducted on these five NRGs, demonstrating that they serve as robust prognostic predictors when integrated with beta coefficients in the multivariate Cox regression model. The risk score was calculated as follows: Risk Score = (0.3118) × *TRAF5* + (0.206) × *CAMK2A* + (−0.232) × *CHMP4C* + (0.0474) × *IL33* + (−0.1772) × *IRF9*. Patients were stratified into high-risk and low-risk groups based on their risk scores, using the median risk value as the threshold. Figure 7C depicts the expression levels of these five genes, the distribution of risk scores, and the survival status of patients. An increase in risk score correlates with an elevated risk of mortality and a reduction in survival time, as shown in Figure 7C. High-risk patients with BLCA exhibited poorer OS than low-risk patients, with median survival times of 1.7 years versus 4.5 years, respectively (HR = 1.94, *P* = 1.53e−05). The prognostic signature was further validated by the time-dependent receiver operating characteristic (ROC) curve, as depicted in Figures 7D,E. In BLCA patients, the areas under the ROC curves (AUCs) were 0.679, 0.601, and 0.597, respectively, indicating a high level of predictive accuracy (Figure 7E).

**FIGURE 8:**
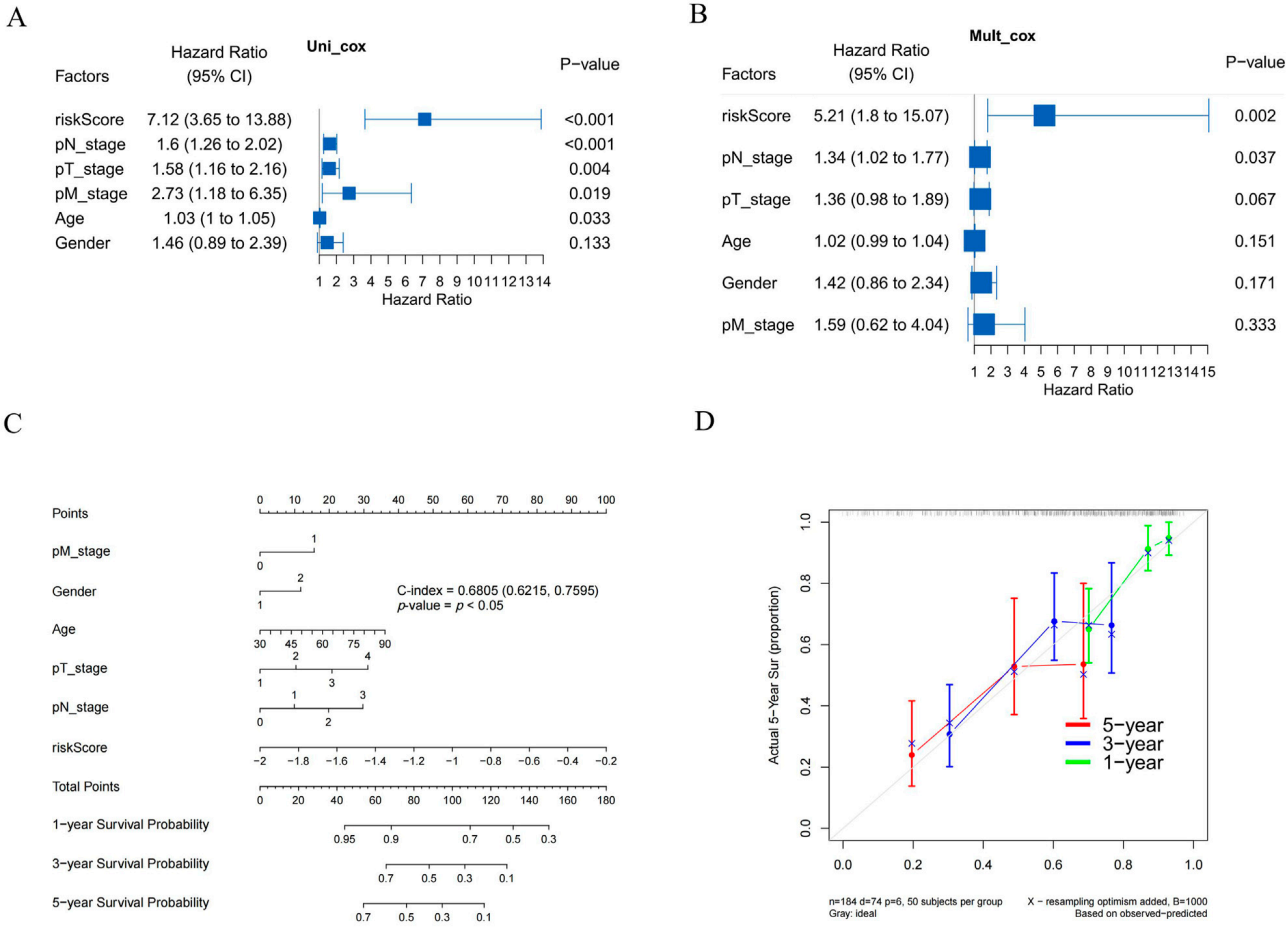
Validation of a prognostic nomogram in BLCA. **(A,B)** Hazard ratios and *P*-values of constituents involved in univariate and multivariate Cox regression and some parameters of five prognostic NRGs in BLCA. **(C)** Nomogram to predict the 1-year, 3-year, and 5-year overall survival rates of BLCA patients. **(D)** Calibration curve for the overall survival nomogram model in the discovery group. A dashed diagonal line represents the ideal nomogram. *P < 0.05, **P < 0.01, and ***P < 0.001. Abbreviations: NRGs, necroptosis-related genes; BLCA, bladder urothelial carcinoma.

To ascertain whether the prognostic signature we developed could independently predict the prognosis of BLCA, we conducted univariate and multivariate Cox regression analyses (Figures 8A,B). The univariate Cox regression analysis identified several clinicopathological factors significantly associated with survival. Notably, the risk score exhibited the highest HR of 7.12 (*P* < 0.001), suggesting a strong correlation with poor prognosis. Other significant associations included pN_stage (HR = 1.60, *P* < 0.001), pT_stage (HR = 1.58, *P* = 0.004), pM_stage (HR = 2.73, *P* = 0.019), and age (HR = 1.03, *P* = 0.033). In the multivariate Cox regression model, the risk score persisted as a significant independent predictor with an HR of 5.21 (*P* = 0.002), while pN_stage (HR = 1.34, *P* = 0.037) demonstrated marginal significance. A nomogram was subsequently constructed, incorporating the risk score and other clinicopathological parameters. The C-index of the nomogram was 0.6805 (*P* < 0.05), indicating moderate predictive accuracy. Within the entire cohort, the predictive nomogram effectively estimated 1-year, 3-year, and 5-year OS rates, aligning closely with ideal predictions.

### Protein expression analysis of CAMK2A, CHMP4C,IL33, IRF9, and TRAF5 in BLCA

**FIGURE 9:**
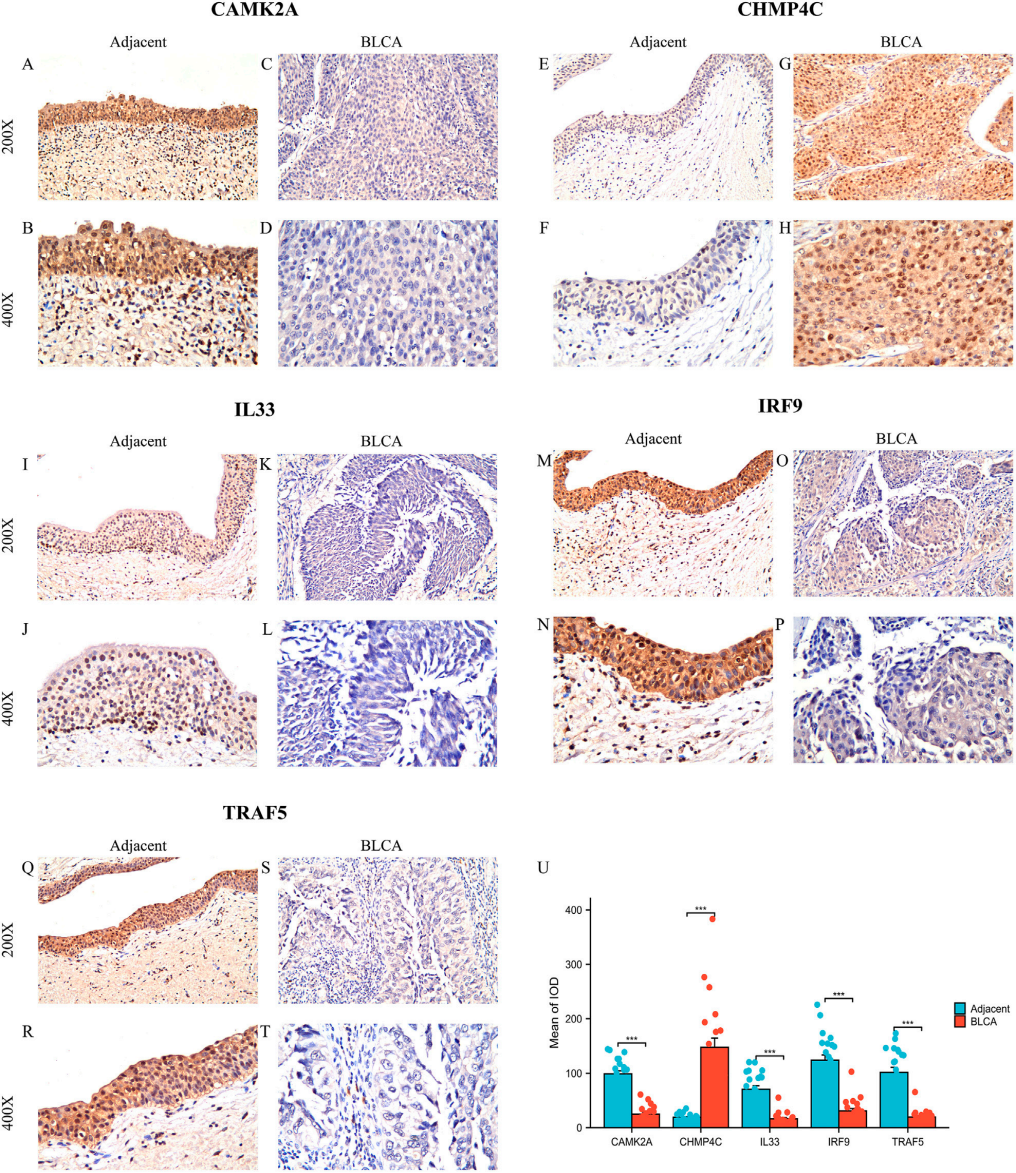
Protein expression analysis of five NGRs. **(A–T)** CAMK2A, CHMP4C, IL33, IRF9, and TRAF5 protein expressions in BLCA tumor tissues and adjacent normal tissues (×200 and ×400 magnification); **(U)** Quantification of immunostains for CAMK2A, CHMP4C, IL33, IRF9, and TRAF5 by IOD analysis. *P < 0.05, **P < 0.01, and ***P < 0.001. Abbreviations: NRGs, necroptosis-related genes; BLCA, bladder urothelial carcinoma; OS, overall survival.

Subsequently, we examined the protein expression of the five NRGs in 22 pairs of BLCA tumor tissues and their corresponding adjacent normal tissues using immunohistochemistry. The IHC staining revealed that CHMP4C protein expression was predominantly localized in the nucleus and cytoplasm of cancer cells (Figures 9G,H), whereas in normal tissues, CHMP4C was either weakly expressed or absent (Figures 9E,F). In contrast, the proteins CAMK2A, IL33, IRF9, and TRAF5 were primarily expressed in normal tissues (Figures 9A,B,I,J,M,N,Q,R) and exhibited reduced expression in tumor tissues (Figures 9C,D,K,L,O,P,S,T). Quantitative immunohistochemical analysis, based on the integrated optical density (IOD) values, corroborated these findings with statistical significance (*P* < 0.001) (Figure 9U). Furthermore, the protein expression levels of these five NRGs were consistent with their mRNA expression profiles in TCGA (Figures 3C,D).

### Correlation between the expression levels of five NRGs and the clinical characteristics of BLCA patients

In light of the distinct prognostic roles exhibited by these five prognostic NRGs in BLCA, we analyzed the expression level variations among these NRGs across different clinical and molecular criteria. Regarding the tumor pathological stage, it was observed that the expression of *CAMK2A* was significantly elevated in patients with stage III and IV BLCA compared to those in stages I and II (*P* < 0.001). Conversely, the expression levels of *CHMP4C* and *IRF9* were lower in stage III and IV patients than in patients in stages I and II (*P* < 0.001) (Tables 2–4). Notably, in terms of the T stage, the mRNA expression of *CAMK2A* was upregulated in patients within the T3 and T4 groups, whereas the mRNA expression of *CHMP4C* was downregulated compared to the T1 and T2 groups (Table 3). Concerning the N stage criterion, *CAMK2A* expression was higher in patients with N1, N2, and N3 BLCA than in those within the N0 group (*P* = 0.003) (Table 2). However, the expression levels of *IRF9* and *TRAF5* were downregulated in patients with N1, N2, and N3 compared to the N0 group (*P* < 0.05) (Tables 5, 6). Regarding age, the expression of *IRF3* and *TRAF5* was upregulated in patients aged ≤70 compared to those aged >70 (*P* < 0.05). Regarding pathological classification, the expression of *CHMP4C* was significantly upregulated in papillary bladder cancer compared to the non-papillary group (*P* < 0.001; Table 3). Conversely, *CAMK2A* expression was significantly lower in papillary bladder cancer than in the non-papillary group (*P* < 0.001; Table 2). As demonstrated in Table 4, most of these prognostic NRG expressions, except for *IL33*, were correlated with tumor stage and lymph node metastasis.

**TABLE 2:** Relationship between *CAMK2A* expression and clinical characteristics of patients with BLCA.

Gene	Characteristics	Total(N)	Odds ratio (OR)	*P*-Value
*CAMK2A*	Age ( ≤ 70 vs. > 70)	408	0.887 (0.599–1.312)	0.549
	Gender (Female vs. Male)	408	1.079 (0.694–1.680)	0.736
	T stage (T1 & T2 vs. T3 & T4)	374	0.289 (0.182–0.454)	<0.001
	N stage (N0 vs. N3 & N2 & N1)	366	0.513 (0.329–0.793)	0.003
	M stage (M0 vs. M1)	207	0.712 (0.207–2.549)	0.586
	Pathologic stage (Stage I & Stage II vs. Stage III & Stage IV)	406	0.292 (0.186–0.451)	<0.001
	Histologic grade (Low Grade vs. High Grade)	405	0.045 (0.002–0.219)	0.003
	Subtype (Non-Papillary vs. Papillary)	403	3.544 (2.284–5.580)	<0.001
	Primary therapy outcome (SD & PR & CR vs. PD)	351	0.501 (0.287–0.859)	0.013
	Radiation therapy (Yes vs. No)	382	0.297 (0.096–0.777)	0.020

**TABLE 3:** Relationship between *CHMP4C* expression and clinical characteristics of patients with BLCA.

Gene	Characteristics	Total(N)	Odds ratio (OR)	P Value
*CHMP4C*	Age ( ≤ 70 vs. > 70)	408	1.173 (0.793–1.737)	0.425
	Gender (Female vs. Male)	408	0.881 (0.566–1.370)	0.574
	T stage (T1 & T2 vs. T3 & T4)	374	2.357 (1.517–3.696)	<0.001
	N stage (N0 vs. N1 & N2 & N3)	366	1.386 (0.902–2.138)	0.138
	M stage (M0 vs. M1)	207	0.620 (0.158–2.121)	0.457
	Pathologic stage (Stage I & Stage II vs. Stage III & Stage IV)	406	2.174 (1.424–3.345)	<0.001
	Histologic grade (Low Grade vs. High Grade)	405	2.606 (1.036–7.438)	0.052
	Subtype (Non-Papillary vs. Papillary)	403	0.477 (0.311–0.728)	<0.001
	Primary therapy outcome (SD & PR & CR vs. PD)	351	1.179 (0.694–2.008)	0.542
	Radiation therapy (Yes vs. No)	382	0.884 (0.360–2.148)	0.784

**TABLE 4:** Relationship between *IL33* expression and clinical characteristics of patients with BLCA.

Gene	Characteristics	Total(N)	Odds ratio (OR)	*P*-value
*IL33*	Age ( ≤ 70 vs. >70)	408	0.887 (0.599–1.312)	0.549
	Gender (Female vs. Male)	408	1.392 (0.894–2.176)	0.144
	T stage (T1 & T2 vs. T3 & T4)	374	0.689 (0.445–1.063)	0.093
	N stage (N0 vs. N3 & N2 & N1)	366	1.062 (0.691–1.632)	0.782
	M stage (M0 vs. M1)	207	3.981 (0.994–26.551)	0.082
	Pathologic stage (Stage I & Stage II vs. Stage III & Stage IV)	406	0.752 (0.495–1.140)	0.181
	Histologic grade (Low Grade vs. High Grade)	405	0.480 (0.178–1.179)	0.121
	Subtype (Non-Papillary vs. Papillary)	403	1.448 (0.954–2.207)	0.083
	Primary therapy outcome (SD & PR & CR vs. PD)	351	0.811 (0.475–1.378)	0.438
	Radiation therapy (Yes vs. No)	382	0.492 (0.183–1.211)	0.135

**TABLE 5:** Relationship between *IRF9* expression and clinical characteristics of patients with BLCA.

Gene	Characteristics	Total(N)	Odds ratio (OR)	*P*-value
*IRF9*	Age ( ≤ 70 vs. >70)	408	1.684 (1.136–2.505)	0.010
	Gender (Female vs. Male)	408	0.927 (0.595–1.441)	0.736
	T stage (T1 & T2 vs. T3 & T4)	374	1.213 (0.787–1.872)	0.383
	N stage (N0 vs. N1 & N2 & N3)	366	1.550 (1.007–2.397)	0.047
	M stage (M0 vs. M1)	207	2.894 (0.810–13.506)	0.125
	Pathologic stage (Stage I & Stage II vs. Stage III & Stage IV)	406	1.571 (1.035–2.395)	0.035
	Histologic grade (Low Grade vs. High Grade)	405	0.900 (0.367–2.182)	0.814
	Subtype (Non-Papillary vs. Papillary)	403	1.070 (0.706–1.624)	0.749
	Primary therapy outcome (SD & PR & CR vs. PD)	351	1.369 (0.806–2.345)	0.248
	Radiation therapy (Yes vs. No)	382	2.079 (0.844–5.597)	0.123

**TABLE 6:** Relationship between *TRAF5* expression and clinical characteristics of patients with BLCA.

Gene	Characteristics	Total(N)	Odds ratio (OR)	*P*-value
*TRAF5*	Age ( ≤ 70 vs. >70)	408	1.553 (1.049–2.307)	0.028
	Gender (Female vs. Male)	408	1.026 (0.659–1.596)	0.910
	T stage (T1 & T2 vs. T3 & T4)	374	1.014 (0.657–1.563)	0.950
	N stage (N0 vs. N1 & N2 & N3)	366	1.550 (1.007–2.397)	0.047
	M stage (M0 vs. M1)	207	0.752 (0.211–2.577)	0.648
	Pathologic stage (Stage I & Stage II vs. Stage III & Stage IV)	406	1.110 (0.733–1.684)	0.622
	Histologic grade (Low Grade vs. High Grade)	405	0.919 (0.374–2.228)	0.850
	Subtype (Non-Papillary vs. Papillary)	403	0.660 (0.433–1.003)	0.052
	Primary therapy outcome (SD & PR & CR vs. PD)	351	1.021 (0.601–1.737)	0.937
	Radiation therapy (Yes vs. No)	382	1.356 (0.560–3.396)	0.502

### Expression levels of five NRGs associated with immune cell infiltration in BLCA

**FIGURE 10:**
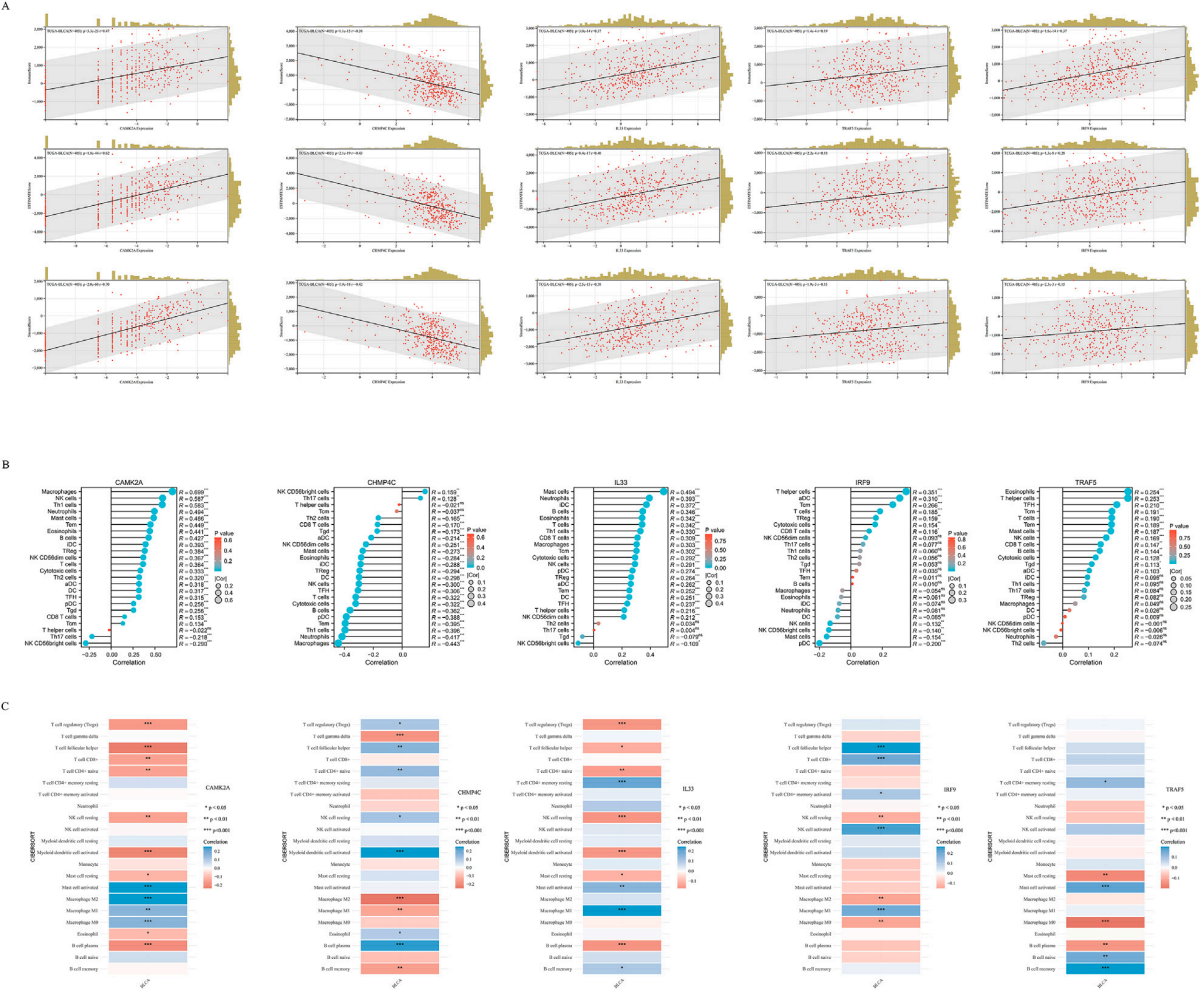
**(A)** The correlation between five prognostic NRGs and tumor microenvironment scores, as determined by the ESTIMATE algorithm, highlighting their association with Immune, Stromal, and ESTIMATE scores in BLCA. **(B)** The relationship between the expression levels of these five prognostic NRGs and immune infiltration in BLCA was analyzed using the ssGSEA algorithm. **(C)** The correlation between the expression levels of the five prognostic NRGs and immune infiltration in BLCA, as assessed by the CIBERSORT algorithm. *P < 0.05, **P < 0.01, and ***P < 0.001. Abbreviations: NRGs, necroptosis-related genes; BLCA, bladder urothelial carcinoma; TILs, tumor-infiltrating lymphocytes.

In BLCA, the expression of five NRGs is linked to clinical characteristics. Tumor-infiltrating lymphocytes serve as an independent predictor of tumor stage, grade, and lymph node status (Ohtani, 2007; Azimi et al., 2012). Utilizing data from TCGA, we examined the relationship between the expression levels of these five prognostic NRGs and immune cell infiltration in BLCA. The ESTIMATE function within the R package was employed to analyze the correlations between Immune Scores, ESTIMATE scores, and Stromal Scores with the expression of these five prognostic NRGs in BLCA. Notably, the expression of *CAMK2A* in BLCA demonstrated a significantly strong positive correlation with the Immune Score (r = 0.47, *P* = 3.3e−23), the ESTIMATE Score (r = 0.62, *P* = 1.8e−44), and the Stromal Score (r = 0.70, *P* = 2.0e−66) (Figure 10A). Similarly, *CHMP4C, IL33, IRF9,* and *TRAF5* also exhibited strong and significant positive correlations with the Immune Score, ESTIMATE Score, and Stromal Score (*P* < 0.001) (Figure 10A). Given the close association of these five NRGs with immune cell infiltration, we further explored their correlation with immune cell infiltration in BLCA using TCGA data.

To elucidate the immunological implications of the five prognostic NRGs in BLCA, we conducted a comprehensive analysis of immune cell infiltration utilizing the ssGSEA algorithm (Figure 10B). Our findings indicated distinct immunomodulatory patterns associated with each NRG. Notably, *CAMK2A* exhibited strong positive correlations with several immune cell types, including macrophages (r = 0.699, *P* < 0.001), neutrophils (r = 0.587, *P* < 0.001), and Th1 cells (r = 0.583, *P* < 0.001), while showing negative correlations with NK CD56 bright cells (r = −0.290, *P* < 0.001) and Th17 cells (r = −0.218, *P* < 0.001). Conversely, *CHMP4C* predominantly demonstrated negative correlations, particularly with macrophages (r = −0.443, *P* < 0.001), neutrophils (r = −0.417, *P* < 0.001), and Th1 cells (r = −0.396, *P* < 0.001), although weak positive associations were noted with NK CD56 bright cells and T helper cells (*P* < 0.01). *IL33*, on the other hand, showed selective positive correlations, with the most significant effects observed in T helper cells (r = 0.351, *P* < 0.001) and activated dendritic cells (aDCs) (r = 0.310, *P* < 0.001). *TRAF5* demonstrated a unique immunoregulatory profile, evidenced by significant positive correlations with various lymphoid cell populations, including naive B cells (r = 0.21, *P* < 0.001), memory B cells (r = 0.15, *P* < 0.01), regulatory T cells (Tregs; r = 0.21, *P* < 0.001), and CD8^+^ T cells (r = 0.12, *P* < 0.05). In contrast, it showed significant negative correlations with cells of the myeloid lineage, such as M0 macrophages (r = −0.18, *P* < 0.001), M2 macrophages (r = −0.14, *P* < 0.01), and neutrophils (r = −0.15, *P* < 0.01). These findings collectively reveal statistically significant associations (*P* < 0.05) between the five prognostic NRGs and immune cell infiltration patterns in BLCA. To ensure the robustness of these results, we employed the CIBERSORT immune infiltration analysis tool, which confirmed the characterization of the tumor immune microenvironment as derived from ssGSEA, thereby validating the consistency of our observations across different analytical methodologies (Figure 10C).

### Immune-related gene analysis of NRGs

**FIGURE 11:**
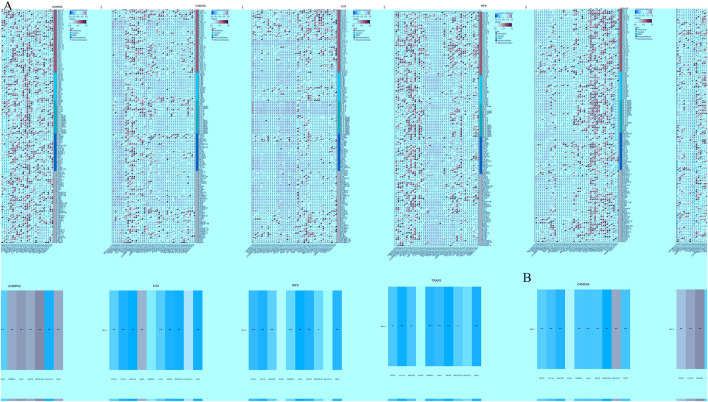
**(A)** The relationship between the expression levels of these five prognostic NRGs and immune-related genes in pan-cancers; **(B)** The relationship between five prognostic NRG expression levels and immune checkpoints in BLCA. *P < 0.05, **P < 0.01, and ***P < 0.001. Abbreviations: NRGs, necroptosis-related genes; BLCA, bladder urothelial carcinoma.

We analyzed the correlations between the mRNA expression levels of five NRGs and immune-related genes, including chemokines, chemokine receptors, major histocompatibility complex (MHC) molecules, immunoinhibitors, and immunostimulators, across 32 distinct cancer types from TCGA (Figure 11A). *CAMK2A* exhibited strong positive correlations across multiple cancer types, with distinct clusters indicating significant associations in specific cancers. *CHMP4C* displayed a diverse correlation pattern, with both positive and negative correlations observed across various cancers. *IL33* was found to have widespread positive correlations in several cancer types, whereas *IRF9* and *TRAF5* demonstrated more heterogeneous correlation profiles. The dendrogram at the bottom of each heatmap illustrates the clustering of gene expression patterns across the different cancer types. Several transcripts associated with immunological checkpoints, such as *SIGLEC15, PDCD1LG2 (PD-L2), TIGIT, PDCD1 (PD-1), CD274 (PD-L1), CTLA4, LAG3*, and *HAVCR2 (TIM3),* play a crucial role in tumor immune evasion. We evaluated the association between the five NRGs and the genes *PDCD1LG2, SIGLEC15, LAG3, TIGIT, CTLA4, CD274, PDCD1*, and *HAVCR2* to explore their potential as predictive biomarkers in BLCA (Figure 11B). Additionally, our analysis revealed a significant positive correlation between *CAMK2A* and *IL33* with *PDCD1LG2, TIGIT, LAG3, CD274, CTLA4, HAVCR2,* and *PDCD1* (*P* < 0.05). Conversely, *CAMK2A* expression exhibited a significant negative correlation with *SIGLEC15* in BLCA. Furthermore, *TRAF5* expression demonstrated a positive correlation with *SIGLEC15, TIGIT, CTLA4,* and *PDCD1* in BLCA (*P* < 0.05). Moreover, the expression levels of *CHMP4C* were significantly negatively correlated with *PDCD1LG2, LAG3, TIGIT, CD274, CTLA4, HAVCR2*, and *PDCD1 (P* < 0.05), as well as with SIGLEC15 (*P* < 0.05).

## Discussion

Necroptosis, a newly recognized form of regulated necrosis triggered by extrinsic apoptotic receptors, is highly inflammatory and can occur when apoptosis is deficient (Boada-Romero et al., 2020; Vantaku et al., 2019). Unlike apoptosis, necroptosis may help overcome tumor cells' resistance to apoptosis and suppress immune responses against cancer (Lee et al., 2019; Mompeán et al., 2018). Despite its potential significance in tumors, our understanding of tumor necroptosis remains limited. BLCA is a highly aggressive malignant tumor with high incidence and recurrence (Seisen et al., 2016). For metastatic BLCA, the effects of conventional treatments are limited. Numerous studies have investigated the association between necroptosis and BLCA, underscoring its significance in prognosis and therapeutic approaches. From a therapeutic perspective, targeting necroptosis represents a promising strategy to circumvent resistance to conventional treatments. For instance, the PKM2 inhibitor Shikonin has demonstrated the ability to overcome cisplatin resistance in BLCA by inducing necroptosis, thereby suggesting an alternative approach to enhance the efficacy of chemotherapy (Wang et al., 2018). Furthermore, the development of tools such as the NecroScore offers a comprehensive evaluation of the impact of necroptosis on responses to immunotherapy and chemotherapy, thereby facilitating personalized treatment planning for patients with BLCA (Zhong et al., 2023).

In this research, we curated a set of 159 NRGs and subsequently identified 25 differentially expressed genes that are functionally implicated in necrotic cell death and extrinsic apoptotic pathways. These pathways specifically include influenza A signaling, NOD-like receptor cascades, and associated biological processes. Through the application of univariate Cox proportional hazards modeling in conjunction with LASSO regression analysis, we identified five NRGs (*CAMK2A, CHMP4C, IL33, IRF9,* and *TRAF5*) with significant prognostic value. These five prognostic NRGs have been shown to contribute to tumor development progression. Specifically, *CHMP4C, IRF9,* and *TRAF5* may function as tumor suppressors. Conversely, *CAMK2A* and *IL33* may facilitate the progression of BLCA.

*CAMK2A* is recognized for its involvement in various cellular processes, including tumor initiation and progression. In the context of lung adenocarcinoma, *CAMK2A* has been demonstrated to support tumor-initiating cells by upregulating SOX2 through the phosphorylation of EZH2, indicating a potential mechanism by which it may also affect bladder cancer (Wang et al., 2020). *CHMP4C*, a component of the endosomal sorting complex required for transport III (ESCRT-III), has been implicated in the progression of several cancers, such as pancreatic and prostate cancer. In pancreatic cancer, *CHMP4C* facilitates progression by inhibiting necroptosis via the RIPK1/RIPK3/MLKL pathway, thereby emphasizing its role in cell survival and proliferation (Yu et al., 2025). Similarly, in prostate cancer, *CHMP4C* is associated with poor prognosis and malignant progression, suggesting its potential as a therapeutic target (Zhang H. et al., 2023). These findings highlight the significance of *CHMP4C* in cancer biology and its possible relevance to BLCA. Furthermore, the immune microenvironment plays a pivotal role in BLCA progression. *IL-33*, a cytokine integral to the modulation of immune responses, may exert an influence on BLCA through its interactions with immune cells and associated signaling pathways. While research specifically examining *IL-33* in the context of BLCA remains limited, its established role in other cancer types suggests it could play a pivotal role in tumor–immune system interactions (Che et al., 2024). Similarly, *IRF9*, a transcription factor involved in the interferon signaling pathway, emerges as a molecule of interest. Although direct evidence connecting *IRF9* to BLCA is currently sparse, its function in regulating immune responses and potential interactions with other signaling molecules, such as *TRAF5*, could offer valuable insights into its role in cancer progression (Choi et al., 2024). *TRAF5*, a member of the TNF receptor-associated factor family, is recognized for its involvement in signal transduction and immune regulation (Zhang et al., 2025). Its interactions with various signaling pathways may significantly impact the development and progression of BLCA. By leveraging these genes, we constructed a robust prognostic model that effectively stratifies patients into high- and low-risk categories, each associated with distinct survival outcomes. The prognostic signature underwent further validation through a time-dependent ROC curve analysis. In patients with BLCA, the AUCs demonstrated a high degree of predictive accuracy. This model exhibited commendable precision in forecasting patient prognosis.

The complex relationship between necroptosis and immune response has gained significant attention recently. Necroptosis, a type of programmed necrotic cell death, significantly impacts both innate and adaptive immunity, particularly in cancer and infectious diseases (Meier et al., 2024). In cancer, necroptosis can enhance antitumor immunity and serve as a therapeutic target against apoptosis-resistant cancer cells. Research delves into how necroptosis affects immune cell infiltration and immunotherapy results in BLCA, revealing that necroptosis may boost CD8^+^ T cell infiltration and enhance the effectiveness of immune checkpoint inhibitors (Zhong et al., 2023). Key necroptotic regulators like RIPK1, RIPK3, and MLKL are often altered in cancer, allowing cells to evade necroptosis (Martens et al., 2021). However, in some cancers, their expression is increased, highlighting necroptosis’s dual role in tumor progression and metastasis (Özdemir, 2023). The inflammatory response from necroptosis can either support or hinder tumor growth.

Therefore, we investigated the relationship between the expression levels of five prognostic NRGs and immune cell infiltration in BLCA. The expressions of *CAMK2A, CHMP4C, IL33, IRF9,* and *TRAF5* in BLCA were found to have a significantly strong positive correlation with the Immune Score, ESTIMATE Score, and Stromal Score. Specifically, *CAMK2A* exhibited strong positive correlations with several immune cell types, whereas CHMP4C predominantly showed negative correlations. *IL33* demonstrated selective positive correlations, with the most pronounced effects observed in T helper cells and aDCs. *TRAF5* displayed a distinctive immunoregulatory profile, characterized by significant positive correlations with various lymphoid cell populations, while also showing significant negative correlations with cells of the myeloid lineage. We also identified significant correlations between the mRNA expression of five NRGs and immune-related genes, including chemokines, chemokine receptors, MHC molecules, immunoinhibitors, and immunostimulators. Our research has the potential to enhance understanding of immunotherapy strategies for BLCA. This study revealed that the five prognostic NRGs involved in carcinogenic processes may be influenced by tumor immune evasion and antitumor immunity.

It is important to acknowledge the limitations of our research. First, an independent dataset should be utilized to validate the necroptosis-related prognostic signature. Additional *in vivo* and *in vitro* studies are warranted to corroborate these findings.

In conclusion, a novel necroptosis-related gene signature, comprising five genes (*CAMK2A, CHMP4C, IL33, IRF9,* and *TRAF5*), has been developed to predict prognosis in patients with BLCA. The expression of these five prognostic NRGs in BLCA was validated through immunohistochemistry. Additionally, our study revealed that these NRGs may play a pivotal role in BLCA carcinogenesis by modulating tumor immune cell infiltration and the expression of immunological checkpoints. Nonetheless, further fundamental research and clinical trials are necessary to advance this field.

## Data Availability

All the datasets were retrieved from the publishing literature, so it was confirmed that all written informed consent was obtained. We obtained raw counts of RNA-sequencing data and corresponding clinical information of tumor tissues and adjacent tissues from 33 types of cancer via The Cancer Genome Atlas (TCGA) dataset and Genotype-Tissue Expression (GTEX) databases (https://tcga-pancan-atlas-hub.s3.us-east-1.amazonaws.com/download/EB%2B%2BAdjustPANCAN_IlluminaHiSeq_RNASeqV2.geneExp.xena.gz; Full metadata).

## Glossary

aDCs: activated dendritic cells
AUC: area under the curve
BLCA: bladder urothelial carcinoma
BP: biological process
CC: cellular component
CI: confidence interval
DAMP: damage-associated molecular pattern
DC: dendritic cell
DDR: DNA repair defect
DEG: differentially expressed gene
DFS: disease-free survival
DSS: disease-specific survival
FC: fold change
FGFR: fibroblast growth factor receptor
GO: Gene Ontology
GTEx: genotype-tissue expression
HR: hazard ratio
ICI: immune checkpoint inhibitor
IOD: integrated optical density
KEGG: Kyoto Encyclopedia of Genes and Genomes
KM: Kaplan–Meier
LASSO: least absolute shrinkage and selection operator
MF: molecular function
MIBC: muscle-invasive bladder cancer
MLKL/pMLKL: mixed lineage kinase domain-like protein
NK cell: natural killer cell
NRG: necroptosis-related gene
OS: overall survival
PFS: progression-free survival
PR: progesterone receptor
RIPK1/RIPK3: receptor-interacting serine/threonine protein kinase 1/3
ROC: receiver operating characteristic
SNP: single-nucleotide polymorphism
TCGA: The Cancer Genome Atlas
TIGER: Tumor Immunotherapy Gene Expression Resource
TIL: tumor-infiltrating lymphocyte
TIME: tumor immune microenvironment
TME: tumor microenvironment
TMT: three-step therapy
TNF: tumor necrosis factor
Treg: T cell regulator
TURBT: transurethral resection of bladder tumor

## References

[B1] Ascione C. M. Napolitano F. Esposito D. Servetto A. Belli S. Santaniello A. (2023). Role of FGFR3 in bladder cancer: treatment landscape and future challenges. Cancer Treat. Rev. 115, 102530. 10.1016/j.ctrv.2023.102530 36898352

[B2] Azimi F. Scolyer R. A. Rumcheva P. Moncrieff M. Murali R. McCarthy S. W. (2012). Tumor-infiltrating lymphocyte grade is an independent predictor of sentinel lymph node status and survival in patients with cutaneous melanoma. J. Clin. Oncol. 30 (21), 2678–2683. 10.1200/JCO.2011.37.8539 22711850

[B3] Barrett T. Wilhite S. E. Ledoux P. Evangelista C. Kim I. F. Tomashevsky M. (2013). NCBI GEO: archive for functional genomics data sets--update. Nucleic Acids Res. 41 D991–D995. 10.1093/nar/gks1193 23193258 PMC3531084

[B4] Bertheloot D. Latz E. Franklin B. S. (2021). Necroptosis, pyroptosis and apoptosis: an intricate game of cell death. Cell. Mol. Immunol. 18 (5), 1106–1121. 10.1038/s41423-020-00630-3 33785842 PMC8008022

[B5] Boada-Romero E. Martinez J. Heckmann B. L. Green D. R. (2020). The clearance of dead cells by efferocytosis. Nat. Rev. Mol. Cell Biol. 21 (7), 398–414. 10.1038/s41580-020-0232-1 32251387 PMC7392086

[B6] Che K. Li J. Chen Z. Li Q. Wen Q. Wang C. (2024). IL-33 in cancer immunotherapy: pleiotropic functions and biological strategies. Cytokine Growth Factor Rev. 10.1016/j.cytogfr.2024.11.005 39638672

[B7] Chen B. Khodadoust M. S. Liu C. L. Newman A. M. Alizadeh A. A. (2018). Profiling tumor infiltrating immune cells with CIBERSORT. Methods Mol. Biol. Clifton, N.J. 1711, 1711243–1711259. 10.1007/978-1-4939-7493-1_12 PMC5895181 29344893

[B8] Chen H. Zhang Y. Chen X. Xu R. Zhu Y. He D. (2023). Hypoxia is correlated with the tumor immune microenvironment: potential application of immunotherapy in bladder cancer. Cancer Med. 12 (24), 22333–22353. 10.1002/cam4.6617 38063246 PMC10757107

[B9] Chen J. Kos R. Garssen J. Redegeld F. (2019). Molecular insights into the mechanism of necroptosis: the necrosome as a potential therapeutic target. Cells 8 (12), 1486. 10.3390/cells8121486 31766571 PMC6952807

[B10] Chen Y. Feng Y. Yan F. Zhao Y. Zhao H. Guo Y. (2022). A novel immune-related gene signature to identify the tumor microenvironment and prognose disease among patients with oral squamous cell carcinoma patients using ssGSEA: a bioinformatics and biological validation study. Front. Immunol. 13, 13922195. 10.3389/fimmu.2022.922195 PMC9351622 35935989

[B11] Choi S. Bae H. G. Jo D. G. Kim W. Y. (2024). The role of IRF9 upregulation in modulating sensitivity to olaparib and platinum-based chemotherapies in breast cancer. Genes (Basel) 15 (7), 959. 10.3390/genes15070959 39062738 PMC11276373

[B12] Ding C. Yu Z. Zhu J. Li X. Dai M. QiangHe (2022). Construction and validation of a necroptosis-related gene signature for predicting prognosis and tumor microenvironment of pancreatic cancer. Dis. Markers 2022, 20229737587. 10.1155/2022/9737587 PMC9214653 35756487

[B13] Dyrskjøt L. Hansel D. E. Efstathiou J. A. Knowles M. A. Galsky M. D. Teoh J. (2023). Bladder cancer. Nat. Rev. Dis. Prim. 9 (1), 58. 10.1038/s41572-023-00468-9 37884563 PMC11218610

[B14] Fritsch M. Günther S. D. Schwarzer R. Albert M. C. Schorn F. Werthenbach J. P. (2019). Caspase-8 is the molecular switch for apoptosis, necroptosis and pyroptosis. Nature 575 (7784), 683–687. 10.1038/s41586-019-1770-6 31748744

[B15] Gielecińska A. Kciuk M. Yahya E. B. Ainane T. Mujwar S. Kontek R. (2023). Apoptosis, necroptosis, and pyroptosis as alternative cell death pathways induced by chemotherapeutic agents. Biochim. Biophys. Acta Rev. Cancer 1878 (6), 189024. 10.1016/j.bbcan.2023.189024 37980943

[B16] Goldman M. J. Craft B. Hastie M. Repečka K. McDade F. Kamath A. (2020). Visualizing and interpreting cancer genomics data *via* the xena platform. Nat. Biotechnol. 38 (6), 675–678. 10.1038/s41587-020-0546-8 32444850 PMC7386072

[B17] Guo Y. Zheng Z. Mao S. Yang F. Wang R. Wang H. (2023). Metabolic-associated signature and hub genes associated with immune microenvironment and prognosis in bladder cancer. Mol. Carcinog. 62 (2), 185–199. 10.1002/mc.23475 36250643

[B18] Guo Z. Liu Y. Chen D. Sun Y. Li D. Meng Y. (2025). Targeting regulated cell death: apoptosis, necroptosis, pyroptosis, ferroptosis, and cuproptosis in anticancer immunity. J. Transl. Int. Med. 13 (1), 10–32. 10.1515/jtim-2025-0004 40115032 PMC11921819

[B19] Huang M. Liu L. Zhu J. Jin T. Chen Y. Xu L. (2021). Identification of immune-related subtypes and characterization of tumor microenvironment infiltration in bladder cancer. Front. Cell Dev. Biol. 9, 9723817. 10.3389/fcell.2021.723817 PMC8438153 34532318

[B20] Ito K. Murphy D. (2013). Application of ggplot2 to pharmacometric graphics. CPT Pharmacometrics Syst. Pharmacol. 2 (10), e79. 10.1038/psp.2013.56 24132163 PMC3817376

[B21] Jeong S. H. Kim R. B. Park S. Y. Park J. Jung E. J. Ju Y. T. (2020). Nomogram for predicting gastric cancer recurrence using biomarker gene expression. Eur. J. Surg. Oncol. 46 (1), 195–201. 10.1016/j.ejso.2019.09.143 31564475

[B22] Kamitani R. Tanaka N. Anno T. Murakami T. Masuda T. Yasumizu Y. (2024). Tumor immune microenvironment dynamics and outcomes of prognosis in non-muscle-invasive bladder cancer. Cancer Sci. 115 (12), 3963–3972. 10.1111/cas.16333 39394691 PMC11611772

[B23] Kanehisa M. Goto S. (2000). KEGG: kyoto encyclopedia of genes and genomes. Nucleic Acids Res. 28 (1), 27–30. 10.1093/nar/28.1.27 10592173 PMC102409

[B24] Khandakar H. Kaushal S. Seth A. Sahoo R. K. Narwal A. Jangir H. (2025). Comparative evaluation of PD-L1 expression and tumor immune microenvironment in molecular subtypes of muscle-invasive bladder cancer and its correlation with survival outcomes. Am. J. Clin. Pathol. 163 (5), 708–722. 10.1093/ajcp/aqae176 39805149

[B25] Konala V. M. Adapa S. Aronow W. S. (2022). Immunotherapy in bladder cancer. Am. J. Ther. 29 (3), e334–e337. 10.1097/MJT.0000000000000934 30839322

[B26] Lee S. B. Kim J. J. Han S. A. Fan Y. Guo L. S. Aziz K. (2019). The AMPK-parkin axis negatively regulates necroptosis and tumorigenesis by inhibiting the necrosome. Nat. Cell Biol. 21 (8), 940–951. 10.1038/s41556-019-0356-8 31358971 PMC6679774

[B27] Leow J. J. Bedke J. Chamie K. Collins J. W. Daneshmand S. Grivas P. (2019). SIU-ICUD consultation on bladder cancer: treatment of muscle-invasive bladder cancer. World J. Urol. 37 (1), 61–83. 10.1007/s00345-018-2606-y 30684034

[B28] Li F. Zheng Z. Chen W. Li D. Zhang H. Zhu Y. (2023). Regulation of cisplatin resistance in bladder cancer by epigenetic mechanisms. Drug resist. updat. 68, 68100938. 10.1016/j.drup.2023.100938 36774746

[B29] Liu S. Wang Z. Zhu R. Wang F. Cheng Y. Liu Y. (2021). Three differential expression analysis methods for RNA sequencing: limma, EdgeR, DESeq2. J. Vis. Exp. JoVE 175. 10.3791/62528 34605806

[B30] Lopez-Beltran A. Cookson M. S. Guercio B. J. Cheng L. (2024). Advances in diagnosis and treatment of bladder cancer. BMJ Clin. Res. ed. 384, 384e076743. 10.1136/bmj-2023-076743 38346808

[B31] Luo J. Luo F. Li Q. Liu Q. Wang J. (2024). An immunogenic cell death-related lncRNA signature correlates with prognosis and tumor immune microenvironment in bladder cancer. Sci. Rep. 14 (1), 13106. 10.1038/s41598-024-63852-9 38849410 PMC11161581

[B32] Martens S. Bridelance J. Roelandt R. Vandenabeele P. Takahashi N. (2021). MLKL in cancer: more than a necroptosis regulator. Cell Death Differ. 28 (6), 1757–1772. 10.1038/s41418-021-00785-0 33953348 PMC8184805

[B33] Mayakonda A. Lin D. C. Assenov Y. Plass C. Koeffler H. P. (2018). Maftools: efficient and comprehensive analysis of somatic variants in cancer. Genome Res. 28 (11), 1747–1756. 10.1101/gr.239244.118 30341162 PMC6211645

[B34] Meier P. Legrand A. J. Adam D. Silke J. (2024). Immunogenic cell death in cancer: targeting necroptosis to induce antitumour immunity. Nat. Rev. Cancer 24 (5), 299–315. 10.1038/s41568-024-00674-x 38454135

[B35] Mompeán M. Li W. Li J. Laage S. Siemer A. B. Bozkurt G. (2018). The structure of the necrosome RIPK1-RIPK3 core, a human hetero-amyloid signaling complex. Cell 173 (5), 1244–1253.e10. 10.1016/j.cell.2018.03.032 29681455 PMC6002806

[B36] Ohtani H. (2007). Focus on TILs: prognostic significance of tumor infiltrating lymphocytes in human colorectal cancer. Cancer Immun. 74, 4. 10.1158/1424-9634.DCL-4.7.1 PMC2935759 17311363

[B37] Otręba M. Stojko J. Rzepecka-Stojko A. (2023). Phenothiazine derivatives and their impact on the necroptosis and necrosis processes. A Rev., 492153528. 10.1016/j.tox.2023.153528 37127180

[B38] Özdemir B. H. (2023). Tumor microenvironment: Necroptosis switches the subtype of liver cancer while necrosis promotes tumor recurrence and progression. Exp. Clin. Transpl. 21 (4), 291–298. 10.6002/ect.2021.0457 35297332

[B39] Peng L. (2024). “Necroptosis and autoimmunity,”, 266. Orlando, Fla. 10.1016/j.clim.2024.110313 Clin. Immunol. 266110313 39002793

[B40] Petrelli F. Perego G. Vavassori I. Luciani A. (2022). Neoadjuvant or adjuvant immunotherapy in bladder cancer: biological opportunity or clinical utility. Tumori 108 (5), 510–511. 10.1177/03008916211061604 34806495

[B41] Seisen T. Peyronnet B. Dominguez-Escrig J. L. Bruins H. M. Yuan C. Y. Babjuk M. (2016). Oncologic outcomes of kidney-sparing surgery *versus* radical nephroureterectomy for upper tract urothelial carcinoma: a systematic review by the EAU non-Muscle invasive bladder cancer guidelines panel. Eur. Urol. 70 (6), 1052–1068. 10.1016/j.eururo.2016.07.014 27477528

[B42] Stekhoven D. J. Bühlmann P. (2012). MissForest--non-parametric missing value imputation for mixed-type data. Bioinformatics 28 (1), 112–118. 10.1093/bioinformatics/btr597 22039212

[B43] Tan Z. Chen X. Zuo J. Fu S. Wang H. Wang J. (2023). Comprehensive analysis of scRNA-Seq and bulk RNA-seq reveals dynamic changes in the tumor immune microenvironment of bladder cancer and establishes a prognostic model. J. Transl. Med. 21 (1), 223. 10.1186/s12967-023-04056-z 36973787 PMC10044739

[B44] Tholomier C. Souhami L. Kassouf W. (2020). Bladder-sparing protocols in the treatment of muscle-invasive bladder cancer. Transl. Androl. Urol. 9 (6), 2920–2937. 10.21037/tau.2020.02.10 33457265 PMC7807363

[B45] Vantaku V. Dong J. Ambati C. R. Perera D. Donepudi S. R. Amara C. S. (2019). Multi-omics integration analysis robustly predicts high-grade patient survival and identifies CPT1B effect on fatty acid metabolism in bladder cancer. Clin. Cancer Res. 25 (12), 3689–3701. 10.1158/1078-0432.CCR-18-1515 30846479 PMC6571061

[B46] Wang S. Q. Liu J. Qin J. Zhu Y. Tin V. P. Yam J. (2020). CAMK2A supported tumor initiating cells of lung adenocarcinoma by upregulating SOX2 through EZH2 phosphorylation. Cell Death Dis. 11 (6), 410. 10.1038/s41419-020-2553-6 32483123 PMC7264342

[B47] Wang Y. Hao F. Nan Y. Qu L. Na W. Jia C. (2018). PKM2 inhibitor shikonin overcomes the cisplatin resistance in bladder cancer by inducing necroptosis. Int. J. Biol. Sci. 14 (13), 1883–1891. 10.7150/ijbs.27854 30443191 PMC6231221

[B48] Xu Z. Zhao Y. Zhang Y. Liu X. Song L. Chen M. (2024). Prediction of immunotherapy response of bladder cancer with a pyroptosis-related signature indicating tumor immune microenvironment. Front. Pharmacol. 15, 151387647. 10.3389/fphar.2024.1387647 PMC11231188 38983908

[B49] Ye K. Chen Z. Xu Y. (2023). The double-edged functions of necroptosis. Cell Death Dis. 14 (2), 163. 10.1038/s41419-023-05691-6 36849530 PMC9969390

[B50] Yin J. Yu Y. Huang X. Chan F. K. (2024). Necroptosis in immunity, tissue homeostasis, and cancer. Curr. Opin. Immunol. 89, 89102455. 10.1016/j.coi.2024.102455 39167896

[B51] Yu G. Wang L. G. Han Y. He Q. Y. (2012). clusterProfiler: an R package for comparing biological themes among gene clusters. OMICS 16 (5), 284–287. 10.1089/omi.2011.0118 22455463 PMC3339379

[B52] YuL.GuoQ.LiY.MaoM.LiuZ.LiT. (2025). CHMP4C promotes pancreatic cancer progression by inhibiting necroptosis *via* the RIPK1/RIPK3/MLKL pathway. J. Adv. Res. 10.1016/j.jare.2025.01.040 39870301

[B53] Yu L. Huang K. Liao Y. Wang L. Sethi G. Ma Z. (2024). Targeting novel regulated cell death: ferroptosis, pyroptosis and necroptosis in anti-PD-1/PD-L1 cancer immunotherapy. Cell Prolif. 57 (8), e13644. 10.1111/cpr.13644 38594879 PMC11294428

[B54] Yuan J. Amin P. Ofengeim D. (2019). Necroptosis and RIPK1-mediated neuroinflammation in CNS diseases. Nat. Rev. Neurosci. 20 (1), 19–33. 10.1038/s41583-018-0093-1 30467385 PMC6342007

[B55] Zang X. Song J. Li Y. Han Y. (2022). Targeting necroptosis as an alternative strategy in tumor treatment: from drugs to nanoparticles. J. Control Release 349, 349213–349226. 10.1016/j.jconrel.2022.06.060 35793737

[B56] Zhang F. Chen X. Qiao C. Yang S. Zhai Y. Zhang J. (2025). Exploring the anti-colorectal cancer mechanism of norcantharidin through TRAF5/NF-κB pathway regulation and folate-targeted liposomal delivery. Int. J. Mol. Sci. 26 (4), 1450. 10.3390/ijms26041450 40003916 PMC11855010

[B57] Zhang H. Liu D. Qin Z. Yi B. Zhu L. Xu S. (2023). CHMP4C as a novel marker regulates prostate cancer progression through cycle pathways and contributes to immunotherapy. Front. Oncol., 131170397. 10.3389/fonc.2023.1170397 PMC10301743 37388224

[B58] Zhang J. He X. Hu J. Li T. (2022). Characterization of necroptosis-related molecular subtypes and therapeutic response in lung adenocarcinoma. Front. Genet. 13, 13920350. 10.3389/fgene.2022.920350 PMC9214237 35754848

[B59] Zhang R. Song Y. Su X. (2023). Necroptosis and alzheimer's disease: pathogenic mechanisms and therapeutic opportunities. J. Alzheimer's Dis. JAD 94 (s1), S367–S386. 10.3233/JAD-220809 36463451 PMC10473100

[B60] Zhang T. Wang Y. Inuzuka H. Wei W. (2022). Necroptosis pathways in tumorigenesis. Semin. Cancer Biol. 86 (Pt 3), 32–40. 10.1016/j.semcancer.2022.07.007 35908574 PMC11010659

[B61] Zhang Z. Lin E. Zhuang H. Xie L. Feng X. Liu J. (2020). Construction of a novel gene-based model for prognosis prediction of clear cell renal cell carcinoma. Cancer Cell Int. 2027, 27. 10.1186/s12935-020-1113-6 PMC6986036 32002016

[B62] Zhang Z. Zhang F. Xie W. Niu Y. Wang H. Li G. (2024). Induced necroptosis and its role in cancer immunotherapy. Int. J. Mol. Sci. 25 (19), 10760. 10.3390/ijms251910760 39409087 PMC11477008

[B63] Zhao Y. Main K. Aujla T. Keshavjee S. Liu M. (2023). Necroptosis in organ transplantation: mechanisms and potential therapeutic targets. Cells 12 (18), 2296. 10.3390/cells12182296 37759518 PMC10527210

[B64] Zhong B. Wang Y. Liao Y. Liang J. Wang K. Zhou D. (2023). MLKL and other necroptosis-related genes promote the tumor immune cell infiltration, guiding for the administration of immunotherapy in bladder urothelial carcinoma. Apoptosis 28 (5-6), 892–911. 10.1007/s10495-023-01830-8 37000317 PMC10232593

[B65] Zhu J. Han T. Zhao S. Zhu Y. Ma S. Xu F. (2022). Computational characterizing necroptosis reveals implications for immune infiltration and immunotherapy of hepatocellular carcinoma. Front. Oncol. 12, 12933210. 10.3389/fonc.2022.933210 PMC9301124 35875102

[B66] Zhu T. Wu B. W. (2024). Recognition of necroptosis: from molecular mechanisms to detection methods. Biomed. and Pharmacother. 178, 178117196. 10.1016/j.biopha.2024.117196 39053418

