# Supplementary material for: Construction and immunohistochemical validation of a necroptosis-related prognostic signature in bladder cancer and its association with tumor immune infiltration
Source: Front Genet. 2025 Aug 14;16:1527907. doi: 10.3389/fgene.2025.1527907 (PMC12391097; doi:10.3389/fgene.2025.1527907)
Supplement: Supplementary file 1 [file Table5.pdf]

Table S5 five NRGs associated with prognosis in BLCA.

Genes	p.value	HR	Low 95%CI	High 95%CI
TRAF5	0.01861	0.700601	0.520915	0.94226849
CAMK2A	0.014833	1.452918	1.075791	1.962249086
CHMP4C	0.001133	0.608342	0.45101	0.82055892
IL33	0.027714	1.396098	1.037262	1.879071164
IRF9	0.016529	0.695906	0.517379	0.936035229
