# Supplementary material for: Construction and immunohistochemical validation of a necroptosis-related prognostic signature in bladder cancer and its association with tumor immune infiltration
Source: Front Genet. 2025 Aug 14;16:1527907. doi: 10.3389/fgene.2025.1527907 (PMC12391097; doi:10.3389/fgene.2025.1527907)
Supplement: Supplementary file 2 [file Table4.pdf]

Table S4 25 NRGs associated with prognosis in BLCA.

Genes	p.value	HR	Low 95%CI	High 95%CI
TRAF2	0.108655	0.785577	0.584977	1.0549662
TRAF5	0.01861	0.700601	0.520915	0.9422685
CAMK2A	0.014833	1.452918	1.075791	1.9622491
SLC25A4	0.772144	1.044443	0.77815	1.4018636
VDAC1	0.616734	1.078123	0.803036	1.447444
PYGM	0.652231	1.070026	0.797182	1.4362525
PLA2G4A	0.784863	0.95988	0.715383	1.2879374
PLA2G4B	0.528943	0.909678	0.677498	1.2214268
PLA2G4C	0.354942	1.149497	0.855669	1.544223
ALOX15	0.323837	0.862259	0.642359	1.1574389
PGAM5	0.66941	1.066292	0.794163	1.4316708
PYCARD	0.118224	0.790235	0.588146	1.0617619
IL1B	0.342802	0.867244	0.646155	1.1639807
CHMP4A	0.881542	1.022692	0.761328	1.3737827
CHMP4C	0.001133	0.608342	0.45101	0.8205589
IL1A	0.650765	0.934282	0.696087	1.2539859
IL33	0.027714	1.396098	1.037262	1.8790712
IFNGR2	0.179263	0.816304	0.607033	1.0977208
STAT1	0.722234	1.05525	0.784479	1.4194803
STAT5B	0.963507	1.006904	0.749882	1.35202
IRF9	0.016529	0.695906	0.517379	0.9360352
TICAM2	0.554447	1.093187	0.813568	1.4689097
BID	0.458047	0.894451	0.666204	1.2008985
BAX	0.391938	0.879334	0.65509	1.1803401
BCL2	0.649415	1.070805	0.797278	1.4381714
