# Supplementary material for: Construction and immunohistochemical validation of a necroptosis-related prognostic signature in bladder cancer and its association with tumor immune infiltration
Source: Front Genet. 2025 Aug 14;16:1527907. doi: 10.3389/fgene.2025.1527907 (PMC12391097; doi:10.3389/fgene.2025.1527907)
Supplement: Supplementary file 3 [file Table1.pdf]

Table S1, Clinical characteristics of patients with BLCA.

Characteristic	No.of patients(%)
**n**	**22**
**Histological grade, n (%)**	
Low grade	5(22.7%)
High grade	17 (77.3%)
**Tumor size, n (%)**	
<3.5 cm	7(31.8%)
≥3.5 cm	15 (68.2%)
**Lymph nodes metastasis, n (%)**	
Yes	3 (13.6%)
NO	19(86.4%)
**Distant metastasis, n (%)**	
Yes	2 (9.1%)
NO	20 (90.9%)
**Invading muscularis, n (%)**	
Yes	9 (40.9%)
NO	13 (59.1%)
