# Supplementary material for: Construction and immunohistochemical validation of a necroptosis-related prognostic signature in bladder cancer and its association with tumor immune infiltration
Source: Front Genet. 2025 Aug 14;16:1527907. doi: 10.3389/fgene.2025.1527907 (PMC12391097; doi:10.3389/fgene.2025.1527907)
Supplement: Supplementary file 4 [file Table2.pdf]

Table S2 159 NRGs from KEGG in BLCA				
gene	VDAC3	CHMP3	IFNA16	PARP1
TNF	GLUD2	RNF103-CHMP3	IFNA17	BID
TNFRSF1A	GLUD1	CHMP4B	IFNA21	BAX
TRADD	GLUL	CHMP4A	IFNB1	AIFM1
TRAF2	PYGL	CHMP4C	IFNG	H2AX
TRAF5	PYGM	CHMP6	IFNAR1	H2AC20
RIPK1	PYGB	VPS4B	IFNAR2	H2AC12
BIRC2	MAPK8	VPS4A	IFNGR1	H2AC1
BIRC3	MAPK10	CHMP1B	IFNGR2	H2AW
XIAP	MAPK9	CHMP1A	JAK1	H2AB3
RBCK1	FTH1	CHMP5	JAK2	H2AC8
RNF31	FTL	CHMP7	JAK3	H2AC4
SHARPIN	PLA2G4E	TRPM7	TYK2	MACROH2A2
SPATA2L	PLA2G4A	IL1A	STAT1	MACROH2A1
SPATA2	JMJD7-PLA2G4B	IL33	STAT2	H2AC19
CYLD	PLA2G4B	HMGB1	STAT3	H2AJ
FADD	PLA2G4C	TNFSF10	STAT4	H2AB1
CASP8	PLA2G4D	TNFRSF10A	STAT5A	H2AC17
CFLAR	PLA2G4F	TNFRSF10B	STAT5B	H2AC18
RIPK3	ALOX15	FASLG	STAT6	H2AC11
CYBB	CAPN1	FAS	IRF9	H2AC21
CAMK2A	CAPN2	FAF1	EIF2AK2	H2AZ2
CAMK2D	SMPD1	IFNA1	TLR4	H2AC7
CAMK2B	MLKL	IFNA2	TICAM2	H2AZ1
CAMK2G	PGAM5	IFNA4	TICAM1	H2AC15
SLC25A4	DNM1L	IFNA5	TLR3	H2AC6
SLC25A5	NLRP3	IFNA6	ZBP1	H2AC13
SLC25A6	PYCARD	IFNA7	USP21	H2AC14
SLC25A31	CASP1	IFNA8	SQSTM1	H2AC16
PPID	IL1B	IFNA10	HSP90AA1	H2AB2
VDAC1	CHMP2A	IFNA13	HSP90AB1	PPIA
VDAC2	CHMP2B	IFNA14	TNFAIP3	BCL2
