# Supplementary material for: Construction and immunohistochemical validation of a necroptosis-related prognostic signature in bladder cancer and its association with tumor immune infiltration
Source: Front Genet. 2025 Aug 14;16:1527907. doi: 10.3389/fgene.2025.1527907 (PMC12391097; doi:10.3389/fgene.2025.1527907)
Supplement: Supplementary file 6 [file Table3.pdf]

Table S3 25 NRGs among the DEGs between BLCA and normal samples.

	logFC	AveExpr	P.Value	adj.P.Val
TRAF2	1.290396	5.067204	1.86E-32	3.63E-31
TRAF5	-1.08244	3.022936	3.07E-15	1.71E-14
CAMK2A	-1.73404	0.535933	4.36E-54	3.98E-52
SLC25A4	-1.08195	4.400907	5.84E-15	3.19E-14
VDAC1	1.192255	8.082572	4.45E-31	7.74E-30
PYGM	-3.35549	1.243721	1.09E-67	3.38E-65
PLA2G4A	-1.26752	3.952649	1.41E-08	4.55E-08
PLA2G4B	-2.36098	1.011561	1.30E-40	4.38E-39
PLA2G4C	-1.13012	1.647827	3.48E-13	1.65E-12
ALOX15	1.050454	1.824687	1.29E-05	3.05E-05
PGAM5	1.352183	5.841835	5.72E-37	1.47E-35
PYCARD	1.125961	6.110998	3.97E-10	1.47E-09
IL1B	1.247208	3.500611	9.31E-05	0.000199
CHMP4A	-1.64855	3.068671	4.85E-34	1.05E-32
CHMP4C	2.380967	5.436198	4.47E-28	6.24E-27
IL1A	2.123908	4.018603	7.21E-08	2.16E-07
IL33	-2.57693	3.294134	1.17E-15	6.74E-15
IFNGR2	1.222389	7.148023	9.46E-30	1.47E-28
STAT1	1.146964	6.95579	8.53E-08	2.54E-07
STAT5B	-1.13145	5.267598	5.13E-30	8.22E-29
IRF9	-1.16197	3.138829	4.60E-11	1.84E-10
TICAM2	-1.40961	0.311479	1.46E-57	1.75E-55
BID	1.312588	5.496648	3.70E-27	4.89E-26
BAX	1.167164	6.904073	8.50E-37	2.15E-35
BCL2	-1.1673	2.310971	8.94E-14	4.44E-13
